# Trimetallic Alloys as an Electrocatalyst for Fuel Cells: The Case of Methyl Formate on Pt_3_Pd_3_Sn_2_

**DOI:** 10.1021/acsami.4c11282

**Published:** 2024-10-16

**Authors:** Radhey Shyam Yadav, Diwakar Kashyap, Itay Pitussi, Medhanie Gebremedhin Gebru, Hanan Teller, Alex Schechter, Haya Kornweitz

**Affiliations:** † Department of Chemical Sciences, Ariel University, Ariel 40700, Israel; ‡ Research and Development Centre for Renewable Energy, New Technology Centre, University of West Bohemia, 301 00 Pilsen, Czech Republic

**Keywords:** Density functional theory, Methyl formate, Electro-oxidation, Fuel cells, d-band center

## Abstract

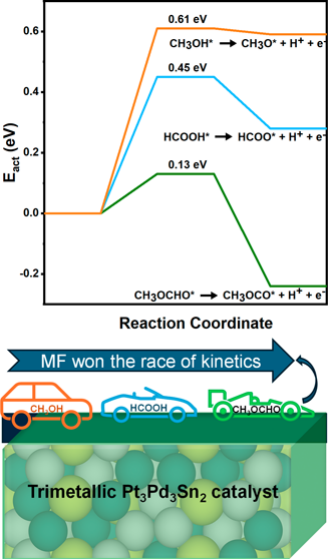

The shift toward renewable energy sources plays a central role in the quest for a circular economy. In this context, methyl formate (MF) has garnered attention as a compelling hydrogen carrier and alternative fuel, because of its remarkable characteristics (energy density, ease of storage and transport, and low boiling point). In this study, DFT calculations supported by online electrochemical mass spectroscopy (OE-MS) were performed to investigate the MF electro-oxidation (MFEO) on Pt_3_Pd_3_Sn_2_ (111). The DFT calculations provide insight into the role of Pt, Pd, and Sn atoms in MFEO. Pt and Pd together provide a preferred active site for initiating MFEO through the O–H bond scission, and Sn plays an essential role in the mitigation of CO through oxygenation or water activation. By comparing the reaction energies and activation barriers for all possible reactions in MFEO, the suggested path necessitates a minimum energy of 0.14 eV to initiate the MFEO. This value was supported by the experimental results, showing that the oxidation wave of MF starts at 0.15 V (70 °C). Density functional theory (DFT) results, supported by OE-MS, indicate that the hydrolysis of MF prior to MFEO is not preferred on Pt_3_Pd_3_Sn_2_ (111) surfaces, although the formation of methanol is plausible via a CH_3_O intermediate. Among the three small organic molecules (SOMs) studied—MF, methanol, and formic acid—MF has the lowest activation energy for the initial bond breaking that starts the whole oxidation process (0.13 eV), compared to formic acid (0.45 eV) and methanol (0.61 eV); thus, MF is the preferred fuel on Pt_3_Pd_3_Sn_2_ (111).

## 1 Introduction

Clean, efficient, and economical energy conversion methods have become a prime focus of public attention in recent years, and their importance is growing.^1,2^ The main reasons are a surge in global energy demands, increased environmental awareness, and reduced dependency on fossil fuels.^1−3^ Polymer electrolyte membrane fuel cells (PEMFCs) are considered an attractive alternative for advancing green energy technologies.^1,4^ Their benefits include low operating temperatures, higher efficiency than internal combustion engines,^5−7^ lower emissions, and eco-friendly acceptable energy conversion products.^6,7^ Furthermore, PEMFCs possess the capability to utilize a wide range of chemical fuels that are either carbon-neutral or produced through renewable energy sources. Among the various fuel options, methyl formate (MF) is a better choice due to various advantages, such as ease of storage in comparison to hydrogen, nontoxicity,^8^ lower crossover through the Nafion proton exchange membrane,^9,10^ and theoretical open circuit voltage (1.23 V)^10^ comparable to methanol (1.20 V)^10^ and dimethyl ether (1.20 V).^11^ MF is an ester having methanolic and formate moieties,^10,12^ The absence of a C–C bond in MF makes its oxidation simpler than ethanol. Moreover, MF is stored as a low boiling point liquid (bp = 32 °C) at ambient storage pressures, which makes its handling and transport in liquid form at room temperature easy, and it can be delivered to fuel cells at high concentration in gaseous form without the usage of active pumping.^9^ MF also has a higher volume charge density (12.5 MC/L) than formic acid (5.11 MC/L) and is comparable to methanol (14.3 MC/L), and it is more cost-effective than formic acid.^10^ Additionally, MF is considered a sustainable fuel because it can be synthesized from the esterification of methanol and formic acid,^13^ which are relatively abundant and can be produced from various renewable sources, including biomass and CO_2_ reduction.^14,15^ Therefore, there has been significant interest in developing direct fuel cells utilizing MF as fuel. The complete oxidation from CH_3_OCHO to CO_2_ would produce eight electrons per MF molecule^16^ (eq 1).
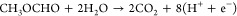
1

Despite the advantages associated with renewable MF fuel, the lack of an efficient anodic catalyst is preventing the wide implementation of direct methyl formate fuel cell (DMFFC). The experimental investigation of MF oxidation has been conducted on various Pt-based bimetallic and trimetallic alloys, including PtRu,^12^ PtPb,^17^ PtSn,^10^ PtPd,^10^ and PtRuPd.^10^ Kucernak et al.^12^ studied the MF oxidation on PtRu/C catalyst and suggested that, during the MF oxidation, the formate component oxidize more easily than the methanolic part. Youn et al.^17^ investigated the electrochemical performance of MF electro-oxidation on PtPb/C catalyst in comparison to the commercial Pt/C and PtRu/C catalysts. In single-cell DMFFC operation, PtPb/C outperforms PtRu/C in maximum power density and open circuit voltage (OCV) for the electrooxidation of MF. Moreover, Youn et al.^10^ conducted an experimental study on the electro-oxidation of MF on PtRuPd/C, comparing its performance with that of Pt/C, PtRu/C, PtSn/C, and PtPd/C catalysts. The findings revealed that the trimetallic PtRuPd/C catalyst exhibited superior activity compared to monometallic or bimetallic catalysts. Additionally, the role of these catalysts in oxidizing the products generated during MF electro-oxidation (MFEO) is investigated. PtPd/C demonstrated outstanding performance in formate oxidation but showed inactivity in methanol oxidation. However, PtSn/C and PtRu/C were observed to promote the oxidation of the methanolic component.^18^ Like other fuels of SOMs, adsorbed carbon monoxide (CO_ad_) is the major poisoning intermediate generated during MF oxidation on platinum-based electrodes.^9,10,12^ Kumar et al.^9^ studied the MF and DME electro-oxidation on the PtPdSn alloy and found that this alloy exhibited superior CO tolerance and enhanced activity compared to the state-of-the-art PtRu alloy. The peak oxidation current for MF on PtPdSn catalyst was two times higher than that on commercial PtRu/C. Further research revealed that Pt_3_Pd_3_Sn_2_ (111) was the most efficient catalyst for SOMs oxidation.^19−21^

Overall, based on the previous investigation, Pt combined with oxophilic materials has demonstrated outstanding performance for the oxidation of the methanolic part and exhibited higher CO tolerance.^10,18^ In the bimetallic and trimetallic catalysts, Sn acts as an oxophilic material, facilitating CO oxidation by offering the OH through the water activation at a lower potential.^10,18−21^ Additionally, Pd has demonstrated superior activity in promoting the oxidation of the formate component.^10,19^ Therefore, we selected the Pt_3_Pd_3_Sn_2_ alloy for the oxidation of MF. As MF is a combination of both the methanolic and formate parts, a catalyst combining Pt, Sn, and Pd is expected to show excellent activity toward its oxidation.

The possibility of MF hydrolysis prior to the MFEO is suggested in the literature.^9,10,12,16,17^ Kumar et al.^9^ could not find significant dissociation of MF on Pt_1_Pd_1_Sn_1_ catalyst in the time scale of the experiment. Also, Barczuk et al.^16^ could not find evidence for hydrolysis using Pt/Pd catalyst. On the other hand, Youn et al.^10^ used PtRu, PtSn, and PtPd, as well as PtRuPd catalysts and found that the dominant reaction is the oxidation of formic acid produced by acid-catalyzed hydrolysis of MF. Acid-catalyzed hydrolysis also occurred to a great extent with the PtPb catalyst.^17^ To get a clear answer to the question of whether hydrolysis occurs prior to electro-oxidation, the full mechanism of the reaction is required. A fundamental understanding of the reaction mechanism is gained by a density functional theory (DFT) study, together with sophisticated experiments, but DFT research has not been done for MFEO on any metal surfaces to date. Such research is necessary to show the importance of MF as a fuel and the advantages of Pt_3_Pd_3_Sn_2_ as a catalyst, revealing the role of each metallic component in this ternary catalyst, elucidating the catalytic electro-oxidation of SOMs in general, and improving the design of anode materials with high activity and selectivity.

Herein, a thorough DFT study of MFEO on Pt_3_Pd_3_Sn_2_ (111) is given. This research aims to investigate various aspects, such as identifying the active adsorption sites, determining the reaction barrier for various steps, analyzing the competing reaction pathways, and confirming the preferred reaction energy path for complete MFEO on the Pt_3_Pd_3_Sn_2_ (111) surface. Online electrochemical mass spectroscopy (OE-MS) was used to validate the intermediates and products found during the electro-oxidation of MF.

## 2 Experimental Section

### 2.1 Computational Details

The DFT-based calculations were performed using the Vienna Ab initio Simulation Package (VASP)^22^ with periodic boundary conditions.^23^ The projected augmented wave (PAW) method was used for describing electron–ion interactions,^24^ and the generalized gradient approximation (GGA)^25^ of Perde, Burke, and Ernzerhof (PBE) was employed to treat exchange-correlation functional.^26^ The plane-wave basis set^27^ was used with a cutoff energy of 400 eV. The details of the calculations were previously described.^21^ The metallic slab model with five layers was used for the surface calculation,^23^ and the Brillouin-zone integrations were performed with a 9 × 9 × 1 Monkhorst–Pack grid for *k*-point sampling.^28^ Initially, to get geometry optimization for the adsorption of all the intermediates on various adsorption sites, a conjugate gradient algorithm^29^ was carried out for all structures until a difference of no more than 1 × 10^–4^ eV in total energy between two successive iterations. Subsequently, the most stable configurations in the best adsorption sites and the transition states were calculated using a convergence criterion of 1 × 10^–5^ eV in total energy between two successive iterations. In all cases, the position of the ions was relaxed, while the cell volume and shape remained constant. van der Waals (VDW) corrections, based on the DFT-D2 by Grimme,^30,31^ and the solvation effect using VASPsol^32^ were considered in all calculations. The zero-point energy (ZPE) was considered for all Gibbs free-energy calculations. The ZPE was calculated by numerical differentiation of forces using a second-order finite difference approach with a step size of 0.015. To save computational resources, ZPE was calculated without considering the solvent effects and the dispersion. The charge transfer between the surface and the adsorbed molecules was calculated using Bader charge analysis.^33^ The transition-state (TS) calculations for activation energy barriers were done by using the climbing image nudged elastic band (CI-NEB) method.^34^ Each calculated transition state was corrected by ZPE calculation (based on ground-state vibrational frequency analysis) to validate the existence of a single imaginary vibrational mode. In computing the transition states, the reactants and products were always adsorbed at the surface. It is assumed that the cleavage of a Z–H (Z = O or C) bond and the release of H^+^ is a two-step process: first, the released hydrogen is adsorbed onto the surface, and then H^+^ + e^–^ is released in an endergonic step. The barrier (*E*_a1_) for the bond cleavage in the first step is determined, and it is assumed that the release of H^+^ + e^–^ occurs without a barrier, unless *E*_a1_ < Δ*G*^0^, Δ*G*^0^ represents the free energy of the two-step process. If *E*_a1_ < Δ*G*^0^, it is assumed that *E*_a_ = Δ*G*^0^, where *E*_a_ is the barrier of the two-step process. All aqueous-phase free energies are calculated relative to the DFT-derived energies of H_2_O (aq), CO_2_ (aq), and H_2_ (aq). An example is given in the Supporting Information (SI). The free energy of adsorption (*G*_ads_), activation energy, and reaction energy are calculated using eqs S5–S7 in the SI. The computational hydrogen electrode model developed by Norskov^35,36^ is utilized to determine the chemical potential of H^+^ + e^–^:
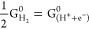
2This equation is based on the definition of zero potential for the standard hydrogen electrode (SHE) under the condition of pH 0, *p* = 1 atm, *T* = 298 K.

As the calculated free energy of adsorption of H* on Pt_3_Pd_3_Sn_2_ (111) surface is −3.91 eV, and ^1^/_2_Δ*G*^0^(H_2_) is −3.22 eV, Δ*G*^0^ of each electro-oxidation step in which H^+^ + e is released, is higher than Δ*G*^0^ of a parallel reaction in which the product is an adsorbed hydrogen (H*) by 0.69 eV. X* means that X is adsorbed at the surface.

The density of states (DOS), projected density of states (PDOS), and the orbital band centers were calculated around the Fermi level, within the energy range of −7.0 to 2.0 eV. The d-band center (ε_d_), p-band center (ε_p_), and s-band center (ε_s_) are calculated by using the following equation:^36^
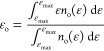
3where ε_o_ represents the band center for the respective orbital (o = d, p, and s), corresponding to the d-band center (ε_d_), p-band center (ε_p_), and s-band center (ε_s_). The term ε is the energy at a given point in PDOS, and *n*_o_(ε) is the orbital-projected density of states for the respective orbitals.

### 2.2 Constructing the Pt_3_Pd_3_Sn_2_(111) Surface

The surface of Pt_3_Pd_3_Sn_2_ was modeled by modifying the fcc bulk structure of (Pt_0.43_Pd_0.57_)_3_Sn, which is based on the AuCu_3_ type crystal structure with a cubic *Pm*3̅*m* (221) space group (ICSD CollCode 105657).^37^ To achieve a stoichiometric ratio representing the Pt_3_Pd_3_Sn_2_ composition, a supercell was first constructed by extending the unit cell along the *x*, *y*, and *z* directions, effectively doubling the original vectors to provide sufficient atomic representation for modifications. Then, the Pt and Pd atoms in the bulk structure were systematically substituted and adjusted in a stoichiometric ratio to represent the Pt_3_Pd_3_Sn_2_ composition, resulting in a configuration containing 12 Pt atoms, 12 Pd atoms, and 8 Sn atoms, as shown in Figure S1a. Subsequently, the enlarged supercell was reduced, using AFLOW software^38^ to a primitive, which is a monoclinic system with space group *c*2/*n* (space group No. 15) (Figure S1b). Finally, the (111) surface orientation was cleaved from this primitive unit cell by using AFLOW software. The resulting surface slab consisted of five atomic layers containing 30 Pt atoms, 30 Pd atoms, and 20 Sn atoms. The top and side views of the Pt_3_Pd_3_Sn_2_ (111) surfaces are shown in Figures S1c ans S1d, respectively.

### 2.3 Experimental Details

A conventional three-electrode cell was used for electrochemical measurements. Graphite rod and Ag/AgCl (3 M KCl) were used as counter and reference, respectively. The Pt_3_Pd_3_Sn_2_/C-coated glassy carbon working electrode was prepared as follows: 5 mg of Pt_3_Pd_3_Sn_2_/C and 28 μL Nafion of Nafon solution (5 wt % in a mixture of lower aliphatic alcohols and water) (20 wt % of the catalyst powder) was first dispersed in a 1 mL of 1:1 distilled water and isopropyl alcohol mixture to prepared the catalyst ink. Following this, an aliquot was taken from this solution and drop-casted on glassy carbon (0.196 cm^2^), such that the loading would be 50 μg cm^–2^ and air-dried. The electrode surface was initially activated by recording a cyclic voltammetry (CV) in an N_2_-saturated 0.5 M of H_2_SO_4_ between 0.05 V and 1.1 V at a rate of 100 mV s^–1^ until a stable voltammogram was achieved. Following this, 0.5 M methyl formate (MF) was added to the electrolyte, and CV was recorded at 10 mV s^–1^.

### 2.4 Online Electrochemical Mass Spectroscopy (OE-MS)

The DFT calculation on the Pt_3_Pd_3_Sn_2_ catalyst was supported by the online gas analysis of moieties formed in the electrooxidation of methyl formate. The procedure catalyst (Pt_3_Pd_3_Sn_2_) preparation and characterization, and the experimental setup for online gas analysis were discussed in detail in an earlier work.^21^ In short, the online gas analysis of methyl formate oxidation was examined in a fuel cell operated in a half-cell mode. The Pt_3_Pd_3_Sn_2_ anode served as a working electrode, and the Pt black cathode served as a counter electrode. A platinum wire touching the Nafion membrane served as a reference electrode. Methyl formate (bp = 32 °C) was supplied to the anode in the gas phase along with carrier gas (argon) at a flow rate of 10 mL min^–1^. The carrier gas was bubbled through a 1.0 M methyl formate aqueous solution and supplied to the anode of the fuel cell. An aqueous solution of methyl formate (1.0 M) was heated at 80 °C and supplied in the gaseous form to the anode via a carrier gas (argon), whereas humidified nitrogen was streamed through the cathode. The background signal was obtained at open circuit potential (OCP), and methyl formate oxidation was examined in the potential range of 0.1–1.0 V vs RHE. The online mass spectroscopy was performed at 70 °C to avoid the condensation of methyl formate oxidation products.

## 3 Results and Discussion

### 3.1 Thermochemistry of Adsorption

**Figure 1:**
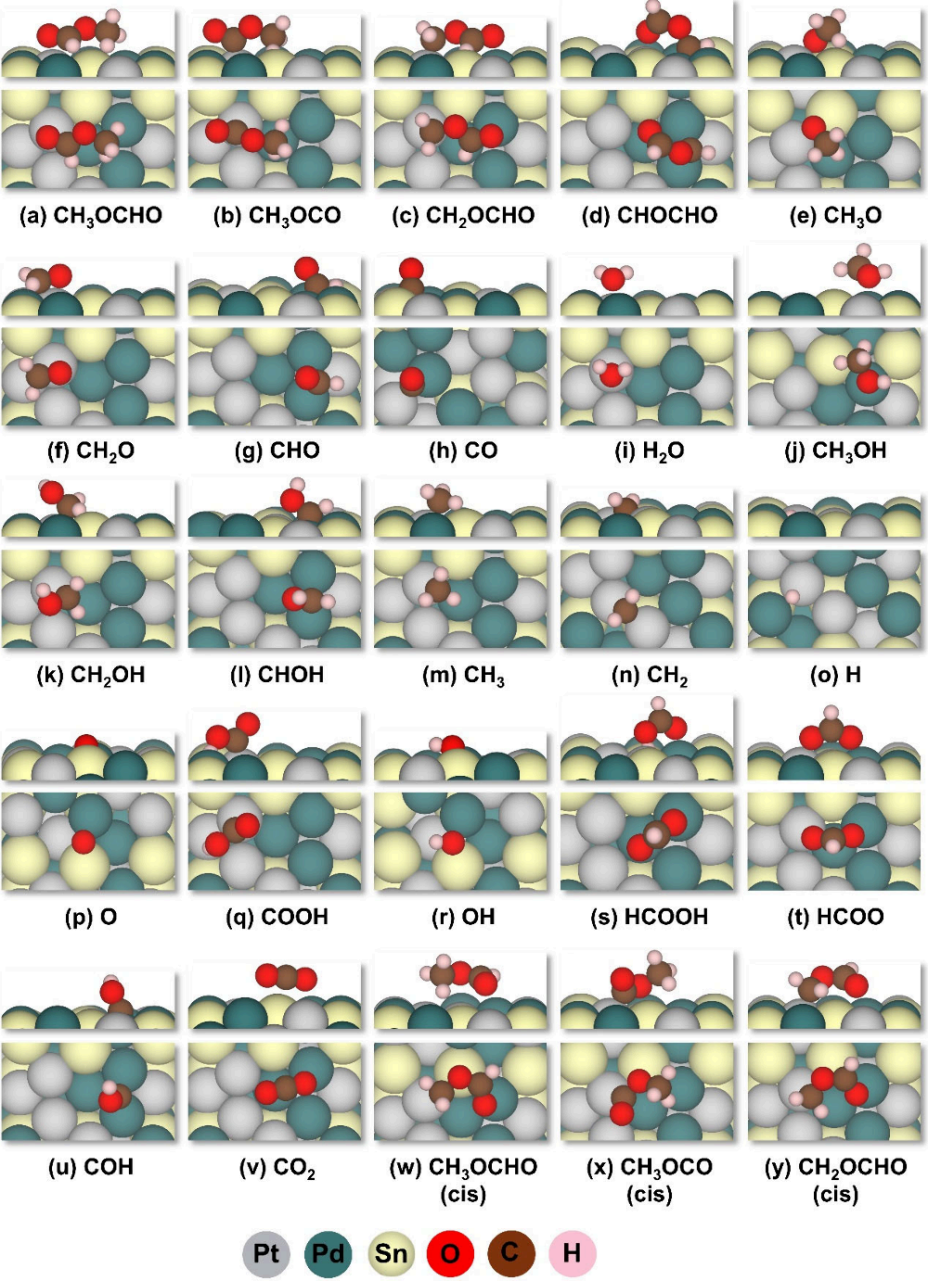
(a–y) Most stable configurations of all the intermediates involved in MFEO.

**Figure 2:**
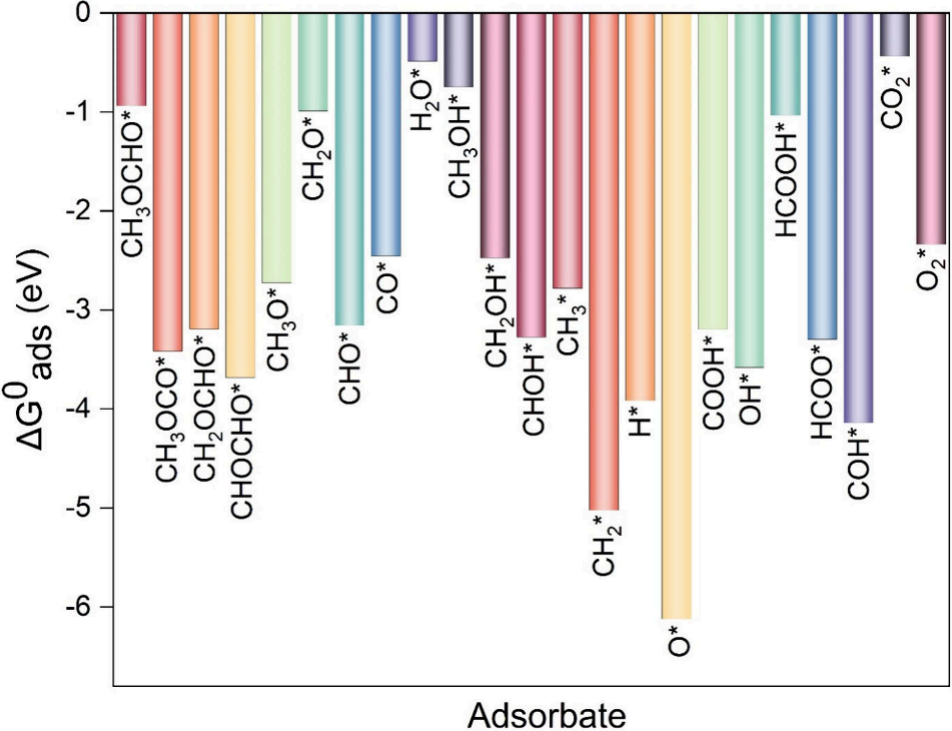
Comparison of adsorption free energies of the most stable configurations of all of the intermediates involved in MFEO.

DFT calculations were performed to study the electro-oxidation of MF on the Pt_3_Pd_3_Sn_2_ (111) surface. The most stable adsorption site for all of the intermediates involved in the MFEO is depicted in Figure 1. The free energy of adsorption for the most stable configuration for all the species involved in MFEO is summarized in Table 1. The comparison between them is depicted in Figure 2. The electronic energy of adsorption for all of the intermediate species on different adsorption sites is given in Table S1. According to the calculations, the most stable adsorption of MF occurs in the trans configuration, which is adsorbed on top of Pt via aldehydic oxygen (Figure 1a), while the methyl component is adsorbed on the Pd atom. Conversely, the cis configuration of MF is adsorbed via its aldehydic oxygen on the top site of the Pd atom, and the methyl part is bonded through C and H atoms on Pt (Figure 1w). The adsorption energy of MF on the Pt_3_Pd_3_Sn_2_ (111) surface is −0.94 eV for trans and −0.73 eV for cis configuration, respectively, meaning that trans-MF is more strongly adsorbed than the cis MF on this ternary alloy. Table S2 shows the charge transfer (CT) between the MF molecule and the surface of Pt_3_Pd_3_Sn_2_ (111) for both trans and cis configurations. The CT from surface to adsorbate is 0.10 e for trans-MF and only 0.01 e for cis-MF; since this CT stabilizes the adsorption, it explains the stronger adsorption of trans-MF than cis-MF. The largest CT is to atoms C2 and O2 (the definition of this notation is depicted in Table S2) that are above Pt and Pd; for trans-MF, the values are 0.12 e and −0.07 e, and for cis-MF, the values are 0.07 e and −0.04 e for C2 and O2, respectively. These values indicate that the aldehydic part of MF plays a major role in the adsorption of MF on this ternary alloy. According to the Bader charge analysis, the charge transfer (CT) is significant from Pt and Pd to carbon and aldehydic oxygen atoms. However, no CT is observed between Sn and the central oxygen atom, as shown in Table S2, this is due to the considerable distance between Sn and the oxygen atom, which is 3.66 Å for trans and 3.41 Å for cis configuration. This distance indicates that the Sn is not directly involved in the MF adsorption for both trans and cis configurations; the adsorption is via the oxygen and carbon of the aldehydic part of the MF.

**Table 1:** Free Energy of Adsorption for All of the Species Involved in MFEO

SN	species	adsorption site	*E*_ads_ (eV)	ZPE (eV)
1	CH_3_OCHO*	Pt–Pd (atop)	–0.94	1.62
2	CH_3_OCO*	Pt (atop)	–3.42	1.32
3	CH_2_OCHO*	Pt–Pd (atop)	–3.19	1.34
4	CHOCHO*	Pd–Pd (atop)	–3.68	1.02
5	CH_3_O*	Pt–Sn (bridge)	–2.73	1.08
6	CH_2_O*	Pt (atop)	–0.99	0.77
7	CHO*	Pd–Pd (bridge)	–3.15	0.44
8	CO*	Pt–Pt (bridge)	–2.45	0.21
9	H_2_O*	Pt (atop)	–0.49	0.62
10	CH_3_OH*	Pd (atop)	–0.75	1.41
11	CH_2_OH*	Pt (atop)	–2.47	1.09
12	CHOH*	Pd–Pd (bridge)	–3.27	0.79
13	CH_3_*	Pt (atop)	–2.78	0.94
14	CH_2_*	Pd–Pt (bridge)	–5.02	0.66
15	H*	Pd–Pt (bridge)	–3.91	0.22
16	O*	Pd–Sn (bridge)	–6.12	0.06
17	COOH*	Pt (atop)	–3.19	0.60
18	OH*	Pd–Sn (bridge)	–3.58	0.35
19	HCOOH*	Pd (atop)	–1.03	0.90
20	HCOO*	Pd–Pt (bridge)	–3.29	0.62
21	COH*	Pd–Pd (bridge)	–4.14	0.48
22	CO_2_*	Pd (atop)	–0.43	0.30
23	O_2_*	Pd–Pt (bridge)	–2.33	0.12

**Figure 3:**
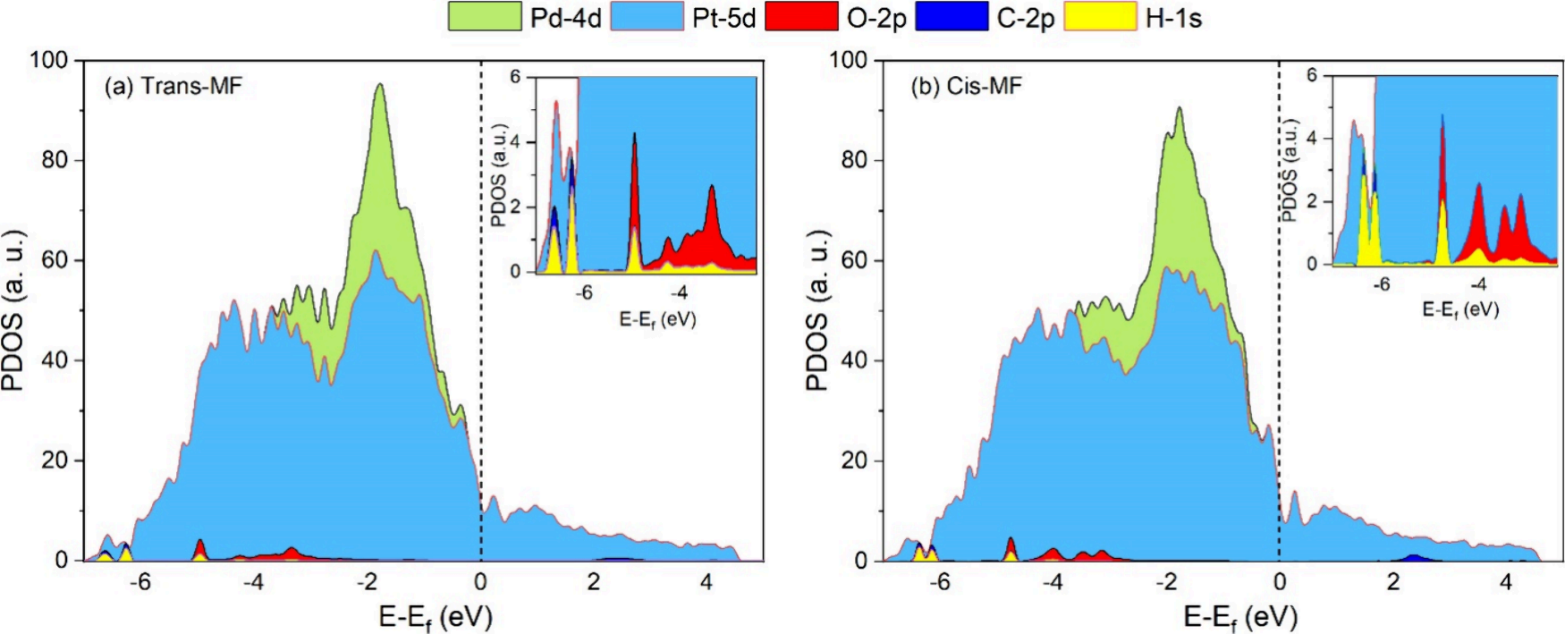
PDOS for the MF adsorption on Pt_3_Pd_3_Sn_2_ (111) alloy: (a) trans configuration and (b) cis configuration.

It is known that the electronic properties affect the adsorption energy significantly. To get an insight into the role of each component in the MF adsorption for trans and cis configuration, we studied the electronic structure of the adsorbed MF on Pt_3_Pd_3_Sn_2_ (111), and the results are shown in Figure 3. The metal surface and the adsorbate form a covalent bond via p-d hybridization. The p and d bands in the partial density of states (PDOS) overlap near the Fermi level (ε_f_), while the d-band center (DBC) and p-band center (PBC) are at different positions. Δ_dP_ and Δ_ds_ are the differences between the DBC and the PBC or the SBC (s-band center); these values are indicators of the overlapping; smaller values mean better overlap and stronger bonds. Therefore, they can be used to estimate the strength of the adsorption.
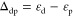
4
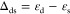
5ε_d_, ε_p_, and ε_s_, are DBC, PBC, and SBC, respectively. The values of ε_d_, ε_p_, and ε_s_ and their differences are summarized in Table S3, and the partial density of states (PDOS) for the trans and cis configuration of MF adsorption are shown in Figures 3a and 3b, respectively.

In the adsorption of MF on Pt_3_Pd_3_Sn_2_ (111), the Δ_dp_ for Pt 5d and C 2p is 0.49 eV for trans and 0.59 eV for the cis configuration; the lower Δ_dp_ for the trans configuration indicates that the bonding through the C atom on Pt contributes to the adsorption, consistent with the former CT values, as shown in Table S2. Similarly, the Δ_dp_ values for Pd 4d and C 2p are smaller for the trans configuration (0.73 eV) than for the cis (0.83 eV), indicating stronger bonding of the trans configuration on the Pd site. The Δ_dp_ values for Pt 5d, or Pd 4d, and O 2p are almost the same for both trans (0.99 and 1.24 eV) and cis (0.97 and 1.21 eV) configurations, meaning that the difference in the adsorption of trans-MF and cis-MF is not attributed to the O 2p orbital. The changes in Pt 5d or Pd 4d, and H 1s exhibit a similar pattern to the alterations in C 2p, with the trans configuration displaying lower values (2.56 and 2.81 eV) compared to the cis configuration (2.66 and 2.90 eV), showing the stronger orbital overlapping via metal-d and H for trans configurations. The electronic properties support the preferred trans-MF adsorption (−0.94 eV) rather than cis-MF (−0.73 eV).

### 3.2 Determination of the Reaction Pathways

**Scheme 1:**
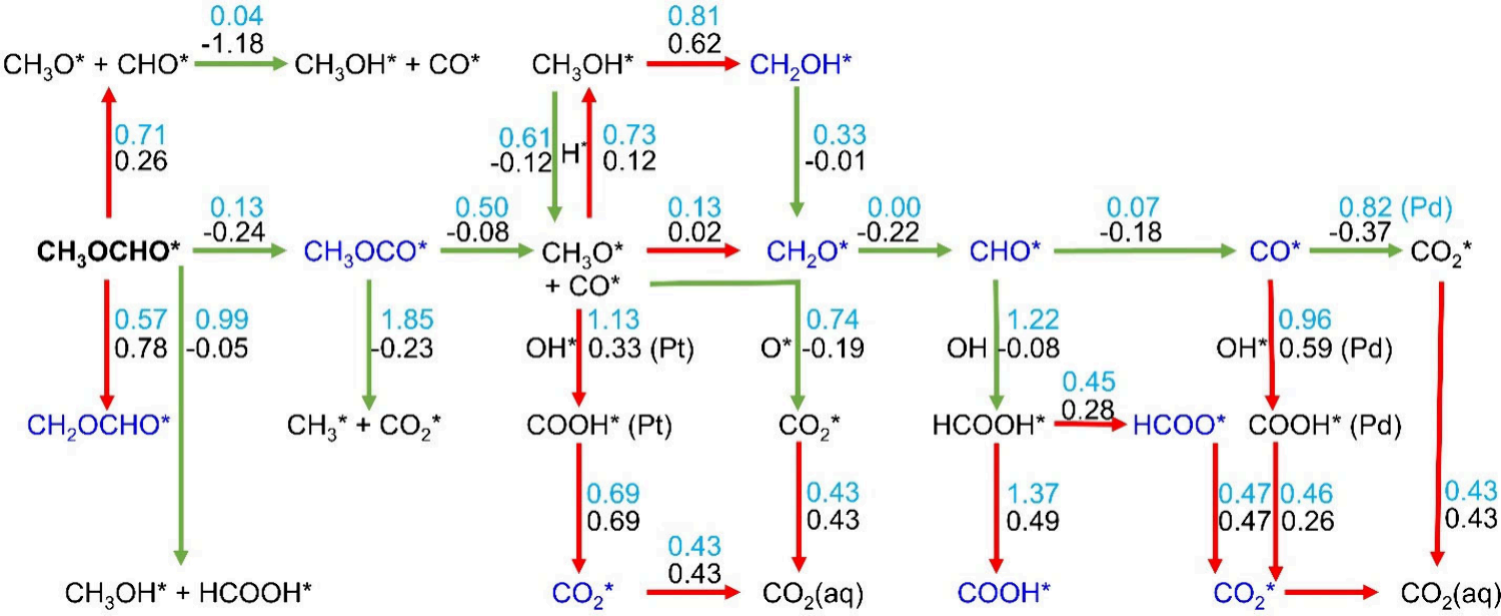
Possible Reaction Pathways of MFEO The green and red color arrows represent the exergonic and endergonic reactions, respectively. The numbers with black and sky-blue colors represent the reaction energy and activation energy (in eV), respectively. Products with blue and black colors show the proton-electron transfer steps and decomposition reactions, respectively.

**Figure 4:**
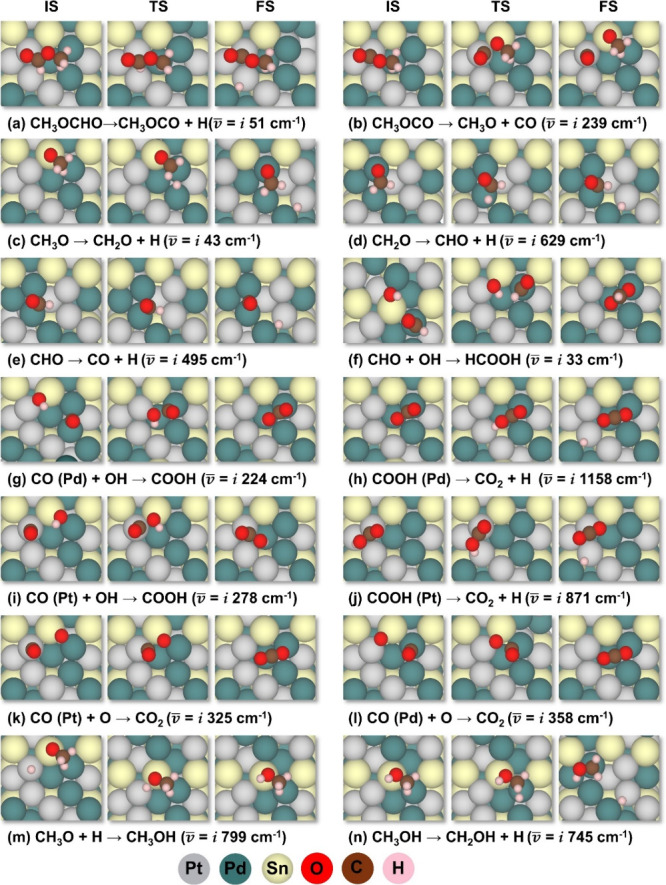
(a–n) Geometries of the initial state (IS), final state (FS), and transition state (TS) involved in the favored reaction of MF electro-oxidation.

All of the intermediates and elementary steps with their thermochemical reaction energies are shown in Scheme S1 and Table S4. In addition to thermochemical energies, consideration of kinetic effects is essential to determine the reaction pathways involved in MFEO. The activation barriers elucidate the actual reaction pathways and the active sites involved in the MFEO. The calculated activation barriers, along with the respective free-energy changes for the various possible steps in the MFEO, are summarized in Table S5; the corresponding reaction steps are depicted in Scheme 1. The geometries of the initial state (IS), final state (FS), and transition state (TS) involved in the preferred reaction steps are shown in Figure 4, and the data for the alternative reactions are given in Figure S2. The preferred pathway is determined according to the lowest barrier in the forward directions in each step.

#### 3.2.1 Initial Bond Cleavage of CH_3_OCHO

Four alternative competing reactions may occur in the initial decomposition of trans-CH_3_OCHO* on Pt_3_Pd_3_Sn_2_ (111) surface: (a) deprotonation from the aldehydic part to form CH_3_OCO* (reaction 6), (b) deprotonation from the methanolic part to form CH_2_OCHO* (reaction 7), (c) C–O bond cleavage (reaction 8) that is followed by reaction 9 to form CH_3_OH* and CO*, an almost barrierless, exergonic process, and (d) hydrolysis of methyl formate (reaction 10).
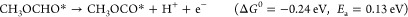
6
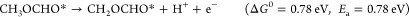
7
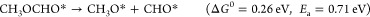
8
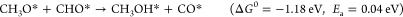
9
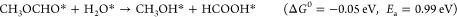
10

The C–H bond scission from the aldehydic part (reaction6), is the most favored reaction, due to the negative Δ*G*^0^ value (−0.24 eV) and the low activation barrier (0.13 eV). This low barrier is attributed to the stronger adsorption of the product^39^ CH_3_OCO* (−3.42 eV) than CH_2_OCHO* (−3.19 eV) on the Pt_3_Pd_3_Sn_2_ (111) surface. In this catalytic event, trans-CH_3_OCHO* is the IS while CH_3_OCO* + H* are the FS, CH_3_OCO* is adsorbed on the top of Pt through the aldehydic C, while the methyl is on the Pd, and H* on the bridge site of Pt–Pd (Figure 4a), this is the adsorption site of H* in all the following steps. Herein, Pt and Pd play a crucial role in initiating the oxidation of MF by providing the active site of moderate adsorption and enhancing the reaction kinetics by lowering the energy barriers. reaction 10 is also exergonic, but its plausibility is lower due to its relatively high barrier (0.99 eV). In previous experimental studies of MFEO, the possibility of hydrolysis was proposed,^9,10,12,16,17^ Since Δ*G*^0^ of reaction 10 is exergonic (−0.05 eV), hydrolysis is thermodynamically plausible. This process is discussed in detail in section 3.4. The configurations of the IS, FS, and the TS of all other reactions are given in Figure S2. These four initial reactions, including their Δ*G*^0^ values and their barriers, are given in Scheme 1.

#### 3.2.2 Decomposition of CH_3_OCO

There are three competing reactions for the dehydrogenation of CH_3_OCO*:
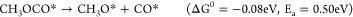
11
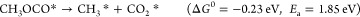
12
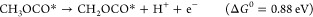
13

The decomposition of CH_3_OCO* to CH_3_O* and CO* (reaction 11) is the most preferred reaction kinetically, rather than C–O bond scission (reaction 12) and C–H bond scission (reaction 13). The CH_3_OCO (reaction 11) is adsorbed via C on Pt. During the decomposition, the C–O bond between CO* and CH_3_O* is elongated, leading to the breaking of this bond, forming CO* that is adsorbed on the top of Pt, and CH_3_O* that is adsorbed on Pd–Sn as FS (Figure 4b). Sn has an essential role in providing the active site for the cleavage of the CH_3_O* and CO* bond during the TS (Figure 4b), resulting in an activation barrier of 0.50 eV, and Δ*G*^0^ of −0.08 eV. The configurations involved in reactions 11 and 12 are presented in Figures 4b and S2c, respectively. Reaction 13 is highly endergonic (0.88 eV); therefore, its barrier was not calculated.

#### 3.2.3 Dehydrogenation of CH_3_O

During the dehydrogenation (CH_3_O* →CH_2_O* + H^+^ + e^–^), the CH_2_O* product is adsorbed on the top site of Pd (Figure 4c). This deprotonation step is almost a thermoneutral (Δ*G*^0^ = 0.02 eV) reaction, with a relatively low activation barrier of 0.13 eV.

#### 3.2.4 Dehydrogenation of CH_2_O

The decomposition of CH_2_O* is depicted in Figure 4d. The dehydrogenation of CH_2_O* (CH_2_O* →CHO* + H^+^ + e^–^) is initiated by H abstraction. The remainder of CHO* is adsorbed at the bridge site of Pd–Pd (Figure 4d); therefore, the released H migrates to the nearby location of the Pt–Pd bridge site. This reaction is barrierless, with an exergonicity of −0.22 eV.

#### 3.2.5 Oxidation of CHO

Two competing reactions may occur during the oxidation of CHO*. It can undergo dehydrogenations to produce CO* (reaction 14) or react with adsorbed OH* (from activated water) to form HCOOH* (reaction 15) via the Langmuir–Hinshelwood mechanism.^40^
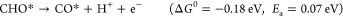
14
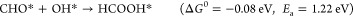
15

The dehydrogenation of CHO* is exergonic (−0.18 eV); it starts by scissoring the C–H bond and forming CO* and H* fragments. CO* is adsorbed vertically on the bridge site of Pd–Pd (Figure 4e). The dehydrogenation of CHO* is the preferred reaction, as it has a significantly lower barrier (0.07 eV), while the formation of formic acid (reaction 15) requires a high activation barrier of 1.22 eV and its exergonicity is lower (−0.08 eV).

### 3.3 CO Removal

The removal of CO* is the most challenging and crucial step on Pt-based alloys during SOMs oxidation.^19,39,41,42^ CO* is adsorbed strongly on the catalyst surface, occupying the active sites and obstructing access for other reactants. During the MFEO, CO* was formed on two different sites: (a) the top site of Pt during the decomposition of CH_3_OCO* (Figure 4b), (b) bridge site of Pd–Pd, in the dehydrogenation of CHO* (Figure 4e). The removal of CO* on the ternary alloy may occur via two possible routes: (a) the formation of COOH* via water activation (reactions 16 and 17, Figures 4g and 4i), and the generation of CO_2_*, according to reactions 18 and 19 (Figures 4h and 4j), (b) through the oxygenation and formation of CO_2_ (see reactions 20 and 21, Figures 4k and 4l).
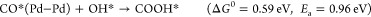
16
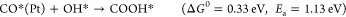
17
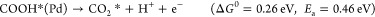
18
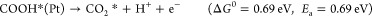
19
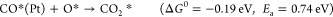
20
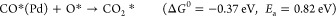
21

**Figure 5:**
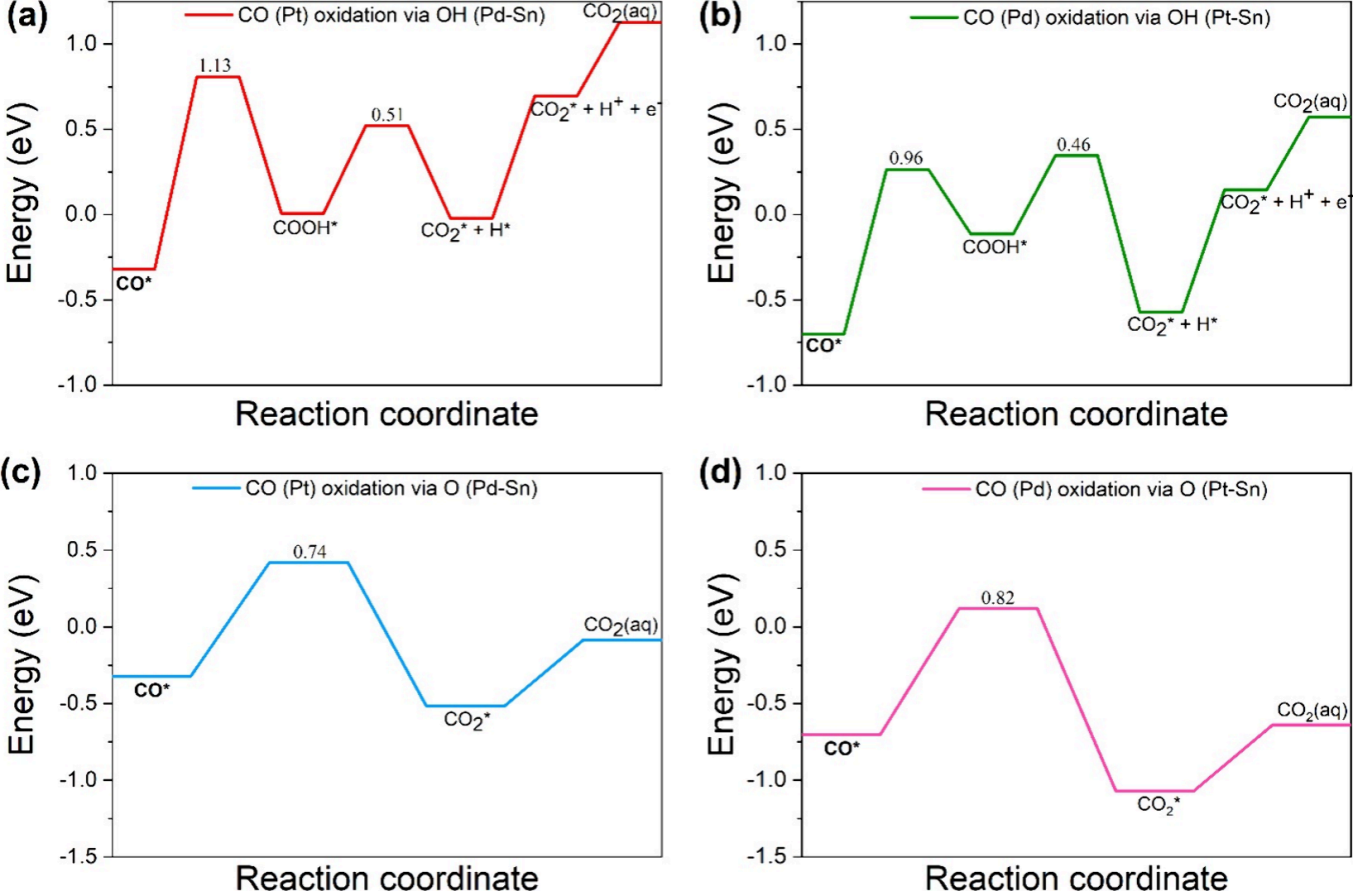
CO* oxidation; (a) via OH* at the Pt site, (b) via OH* at the Pd site, (c) via oxygenation at the Pt site, and (d) via oxygenation at the Pd site.

(a) CO* oxidation via water activation: The removal of CO* from Pt and Pd–Pd sites may occur by water activation, following the Langmuir–Hinshelwood mechanism.^40^ The OH* is formed at the bridge site of Pd–Sn atoms during the water activation. During the oxidation, the CO* from either Pt or Pd–Pd site reacts with the OH* species, and COOH* is formed on the top site of Pt or Pd atoms, respectively. The formation of COOH* takes place in a two-step process: the horizontal diffusion of OH* from the best adsorption site (Pd–Sn) to a neighboring active site of Pt–Sn (site 1) or Pd–Sn (site 2), and then reaction with CO* on Pd–Pd site or on the top site of Pt atom, respectively (Figures 4g and 4i). The adsorption energies at different adsorption sites for O and OH follow the trend: bridge of Pd–Sn (d_Pd–Sn(best ads)_ = 2.88 Å, *E*_ads(OH)_ = −3.58, *E*_ads (O)_ = −6.13 eV) < bridge site of Pt–Sn (site1) (d_Pt–Sn (site 1)_ = 2.71 Å, *E*_ads (OH)_ = −3.45, *E*_ads (O)_ = −6.07 eV) < bridge site of Pd–Sn (site 2) (d_Pd–Sn (site 2)_ = 2.76 Å, *E*_ads (OH)_ = −3.24, *E*_ads (O)_ = −5.73 eV) (Figure S4). The energy required for the OH diffusion to Pt–Sn site (site 1) or Pd–Sn site (site 2) is 0.13 and 0.34 eV, respectively. The COOH* formation at the Pd and Pt sites is endergonic by 0.59 and 0.33 eV, respectively. The activation barrier for the COOH* formation is 0.84 and 0.79 eV for CO* on the Pd–Pd and the Pt site, respectively. The potential energy diagram (PED) for the two-step process in which COOH* is produced is depicted in Figures S3a and S3b. In the next step, dehydrogenation yields CO_2_* (Figures 4h and 4j), in a process with an activation barrier of 0.46 and 0.69 eV on the top site of Pd and the top site of the Pt atom, respectively. Both reactions are endergonic by 0.26 and 0.69 eV, respectively. The PED for these two processes is given in Figures 5a and 5b.
  The removal of CO from the top site of Pt is difficult, as the barrier for this process is high (1.13 eV). Therefore, its oxidation starts with a lateral diffusion to the Pd–Pd bridge site (Figure S5), where it is oxidized via COOH pathway (*E*_a_ = 0.84 eV). This lateral diffusion is only slightly endergonic (0.17 eV) with a barrier of 0.70 eV. The IS, TS, and FS for this lateral diffusion are shown in Figure S5.
(b) CO* oxidation via oxygenation: The CO* removal by oxygenation involves the reaction between CO* and an adsorbed oxygen atom on the surface, resulting in the formation of CO_2_*. The presence of tin oxides, which is verified experimentally,^21^ provides the oxygen for this reaction. Another source of the O* is the Langmuir–Hinshelwood mechanism, wherein an adsorbed O_2_* molecule dissociates into two O* atoms, and O* reacts with CO* to produce CO_2_*. The activation barrier for the dissociation of O_2_* molecule is 0.52 eV with an exergonicity of −1.42 eV. The activation barrier for the oxygenation of CO* on the bridge of Pd–Pd and on the top of the Pt atoms are 0.82 and 0.74 eV, respectively (Figures 4l and 4k). Both reactions are exergonic by −0.37 eV and −0.19 eV, respectively. The PED for these two processes is given in Figures 5c and 5d. The process of CO* removal by oxygenation also occurs in a two-step process: diffusion of O* from the best adsorption site (Figure 1p) to the active site (Figures 4k and 4l), and then reaction with the CO*, as depicted in the PED in Figure S3c,d. As shown in Figure 5, this route is preferred to the CO* oxidation via water activation.

### 3.4 Hydrolysis of MF

**Figure 6:**
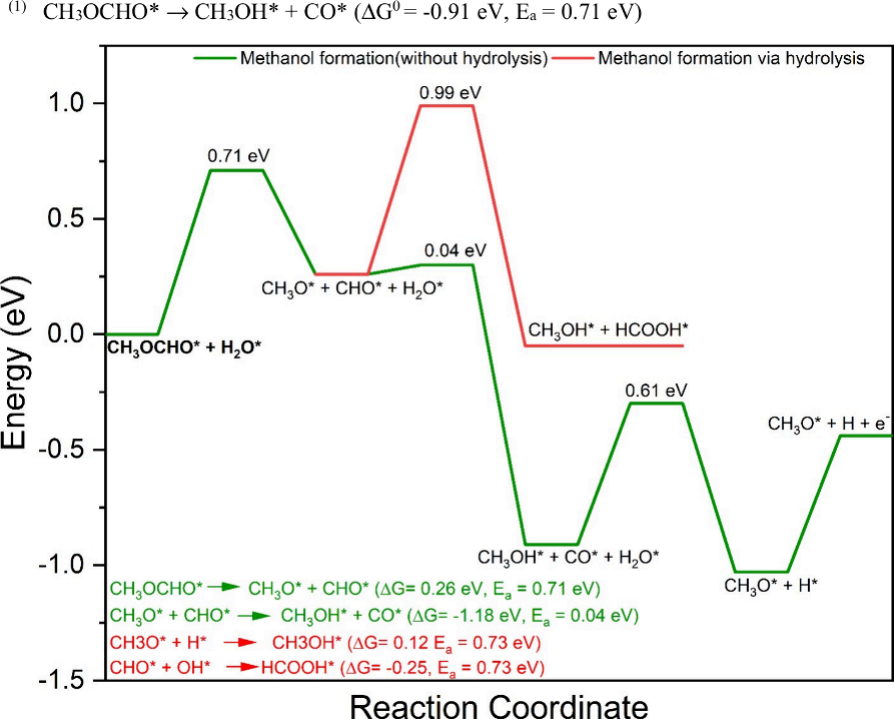
Energetics for the methanol formation on the Pt_3_Pd_3_Sn_2_ (111) surface. The green line shows the methanol formation (without hydrolysis) and the red line represents methanol formation via hydrolysis of MF.

An interesting question is whether methyl formate oxidation (MFO) occurs via chemical hydrolysis (reaction 10) or by electrochemical oxidation through H* abstraction (reaction 6). The lower activation energy of 0.13 eV for the H abstraction from the formate component (reaction 6) suggests that this is the preferred pathway for the MFO. However, reaction 10 shows an exergonic reaction (Δ*G* = −0.05 eV), indicating that MF hydrolysis may also be possible. Hydrolysis is the reaction of MF with activated water to produce formic acid (HCOOH*) and methanol (CH_3_OH*). The activation of water on Pt_3_Pd_3_Sn_2_ (111) was studied previously;^19^ the formation of H* and OH* requires 0.87 eV, because the barrier for splitting water is high. The complete hydrolysis of MF can proceed in two steps: in the first step, the C–O bond in MF is cleaved and CH_3_O* and CHO* (reaction 8) are formed, as shown in Figure S2b, then, CH_3_O* and CHO* react with H* and OH* forming methanol and formic acid (reactions 22 and 23) (see Figures S3e and S3f). The IS, TS, and FS for the methanol formation from CH_3_O* and H* are shown in Figure 4m, and the HCOOH* formation from CHO* (Pt) and OH* is shown in Figure S2n. The PED for complete hydrolysis is shown in Figure 6. The reaction energy and activation barrier required for the hydrolysis on the Pt_3_Pd_3_Sn_2_ (111) surface are −0.05 and 0.99 eV, respectively. This barrier is significantly higher than the barrier of the competing reaction 6 for the H* abstraction (0.13 eV); therefore, hydrolysis is unlikely to occur on the Pt_3_Pd_3_Sn_2_ (111) surface.
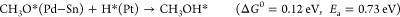
22
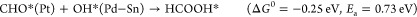
23

### 3.5 Methanol Formation

Although the hydrolysis is not plausible on the Pt_3_Pd_3_Sn_2_ (111) surface, methanol may be produced from CH_3_O* that is formed according to reaction 8 (Δ*G*^0^ = 0.26 eV, *E*_a_ = 0.71 eV). The products of reaction 8 are CH_3_O* and CHO*, and the exchange of the H from CHO* to the CH_3_O* yields CH_3_OH* (reaction 9). This step is almost barrierless (0.04 eV), with an exergonicity of −1.18 eV. Production of CH_3_OH* from MF is summarized in reaction 24.
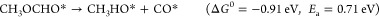
24

### 3.6 Methanol Oxidation

The methanol oxidations may proceed via three initial competing reactions: (a) O–H bond scission (reaction 25), (b) C–H bond scission from the methanolic part (reaction 26), and (c) C–O bond scission to form CH_3_* + OH* (reaction 27).
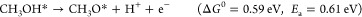
25
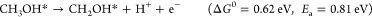
26
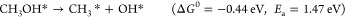
27

Reaction 27 is plausible thermodynamically, because it is exergonic, but this reaction is not plausible kinetically, because its barrier is very high (1.47 eV). Reaction 25 is the most favored reaction due to the relatively low activation barrier; the primary reason for the lower barrier is the stronger adsorption of CH_3_O* (−2.73 eV) than CH_2_OH* (−2.47 eV) on the Pt_3_Pd_3_Sn_2_ (111) surface. The best adsorption site of CH_3_OH* is the top site of the Sn–Pd atoms (Figure 1j). The product CH_3_O* is adsorbed at the same site of Sn–Pd atoms while H* migrates to the top site of the Pt atom (Figure 4m). The reaction is highly endergonic by 0.59 eV and has an activation barrier of 0.61 eV. The oxidation of CH_3_O* proceeded as discussed in Section 3.2.3. The PED for methanol oxidation is shown in Figure 6. The configurations involved in reaction 26 are given in Figure 4n, and in reaction 27 they are given in Figure S2m.

### 3.7 HCOOH* Oxidation

HCOOH is a common product in the electrochemical oxidations of SOMs. It may be formed in MFEO either via hydrolysis or by a combination of CHO* and OH*. There are two possible alternative pathways to oxidize HCOOH*; these pathways are summarized in reactions 28–31.
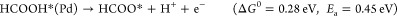
28
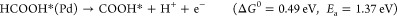
29
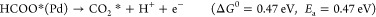
30
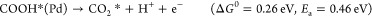
31

The HCOOH* oxidation can start by the O–H bond scission (reaction 28), in which the activation barrier is moderate (0.45 eV), or by C–H bond scission, a process which has a significantly higher barrier (1.37 eV); therefore, the preferred path is via reactions 28 and 30. The HCOOH* molecule is adsorbed on the top site of the Pd atom, HCOO* is adsorbed at the top site of Pt–Pd atom. Subsequent hydrogen abstraction from HCOO* leads to the production of CO_2_*. The second step involves a change in the adsorbed configuration of HCOO*, from vertical to horizontal adsorption, followed by dehydrogenation, leading to the formation of CO_2_* (Figures S2j and S2l)]. The activation barrier and reaction energy for this step is almost similar: 0.47 eV. The COOH formation at the Pd site (reaction 29) exhibits a high barrier of 1.37 eV, which differs from the value at the Pt site^19^ (0.35 eV). The most stable adsorption site for both COOH and CO is on top of Pt. CO* can be formed in an alternative preferred root and occupy the Pt site, preventing the migration of the COOH to this site. Therefore, the COOH* oxidation proceeds via the Pd site only (Figure S2k).

### 3.8 Reaction Paths

**Figure 7:**
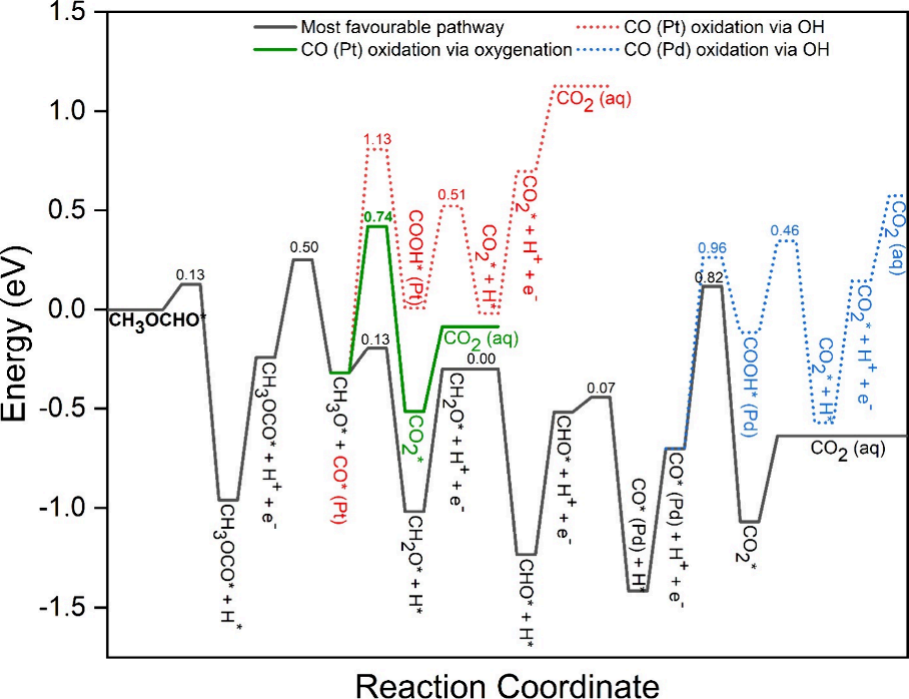
Kinetically favorable pathways for complete oxidation from MF to CO_2_.

**Figure 8:**
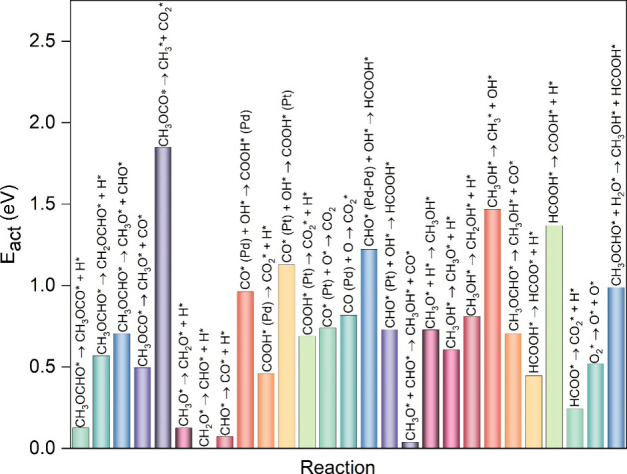
Comparison of the activation energy for all of the steps involved in MFEO.

Figure 7 depicts the energy pathway for MFEO on the Pt_3_Pd_3_Sn_2_ (111) surface, without the effect of an external potential. The energy reference in this study corresponds to the adsorbed MF on the Pt_3_Pd_3_Sn_2_ (111) surface. Four paths are depicted in Figure 7: a black solid line represents the most favorable pathway for the MFEO, while the red and green lines depict the pathway for the CO* oxidations from the top of the Pt site via water activation and oxygenation, respectively. Additionally, a blue line represents the energetics for CO* oxidation via water activation from the Pd bridge site. The preferred initial step in MFEO involves the dehydrogenation of the formate component of adsorbed MF (reaction 6) in its most stable configuration, which has the lowest activation barrier of 0.13 eV (Figure 8). Subsequently, CH_3_OCO* decomposition yields CH_3_O* and CO* as the preferred reaction product. The next step is divided into two pathways: the dehydrogenation of CH_3_O* and the oxidation of CO* at the Pt site, which is the rate-determining step in MFEO. The CO* oxidation via oxygenation (green line) is more plausible, with a lower activation barrier (0.74 eV vs 1.13 eV), compared to the water activation pathway (red line), as depicted in Figure 7. The CH_3_O* pathway proceeds through subsequent H-abstraction to form CHO*. In the next step, two competitive reactions emerge: CO* formation (*E*_a_ = 0.07 eV) from CHO*, which is kinetically preferred over HCOOH* formation (*E*_a_ = 1.22 eV), this preference is attributed to the higher adsorption energy of CO* than HCOOH*. Finally, CO* oxidation from the Pd site is possible via two competitive pathways, the CO* oxidation via oxygenation (black line) being predominant over water activation (blue line), as depicted in Figure 7. In summary, the favored MFEO pathway proceeds via CH_3_OCHO* → CH_3_OCO* → CH_3_O* + CO*(Pt) → CH_2_O → CHO* → CO*(Pd) → CO_2_* → CO_2_ (aq).

This is the preferred path in the presence of air. However, since the fuel-cell experiment was done in an air-free atmosphere without an external source of oxygen, the preferred pathway will follow the blue route, where CO is oxidized on the Pd site via OH. CO that is formed on the Pt site (top) is diffusing to the Pd site (bridge) for oxidation. This diffusion is slightly endergonic by 0.17 eV (*E*_a_ = 0.70 eV).

This study is focused on the MFEO on Pt_3_Pd_3_Sn_2_ (111), but as methanol and HCOOH may be produced during the MFEO (not in the preferred path), their oxidation was studied as well. As methanol and HCOOH are SOMs that are used in fuel cells, the comparison between them and MF is interesting (Figure 8). Initiation of oxidation is a very important step that affects the onset potential; therefore, a comparison of the activation barriers and the reaction energies for breaking the first bond of the various fuels is summarized in Table 2. Our results suggest that the initial bond breaking is more facile for MFEO (reaction 6) on the Pt_3_Pd_3_Sn_2_ (111) surface, compared to the first bond breaking in formic acid (reaction 28) and methanol (reaction 25), primarily due to the lower activation barrier. The first activation barrier for MFEO is only 0.13 eV, while it is 0.45 and 0.61 eV for the formic acid and methanol oxidation, with reaction energies of −0.24, 0.28, and 0.59 eV, respectively. The primary reason may be the adsorption with greater stability of the products CH_3_OCO* (−3.42 eV) than that of HCOO* (−3.29 eV) and CH_3_O* (−2.73 eV). These results indicate that MF is expected to be a better fuel than methanol and FA in a fuel cell in which the Pt_3_Pd_3_Sn_2_ (111) surface is an electrocatalyst.

**Table 2:** Activation Barrier for the First Bond Cleavage on the Pt_3_Pd_3_Sn_2_ (111) Surface for Various Fuels

	methyl formate	formic acid	methanol
*E*_act_ (eV) (first bond cleavage)	0.13 eV	0.45	0.61
Δ*G* (eV)	–0.24	0.28	0.59

### 3.9 Experimental Results

#### 3.9.1 Experimental Electro-oxidation

**Figure 9:**
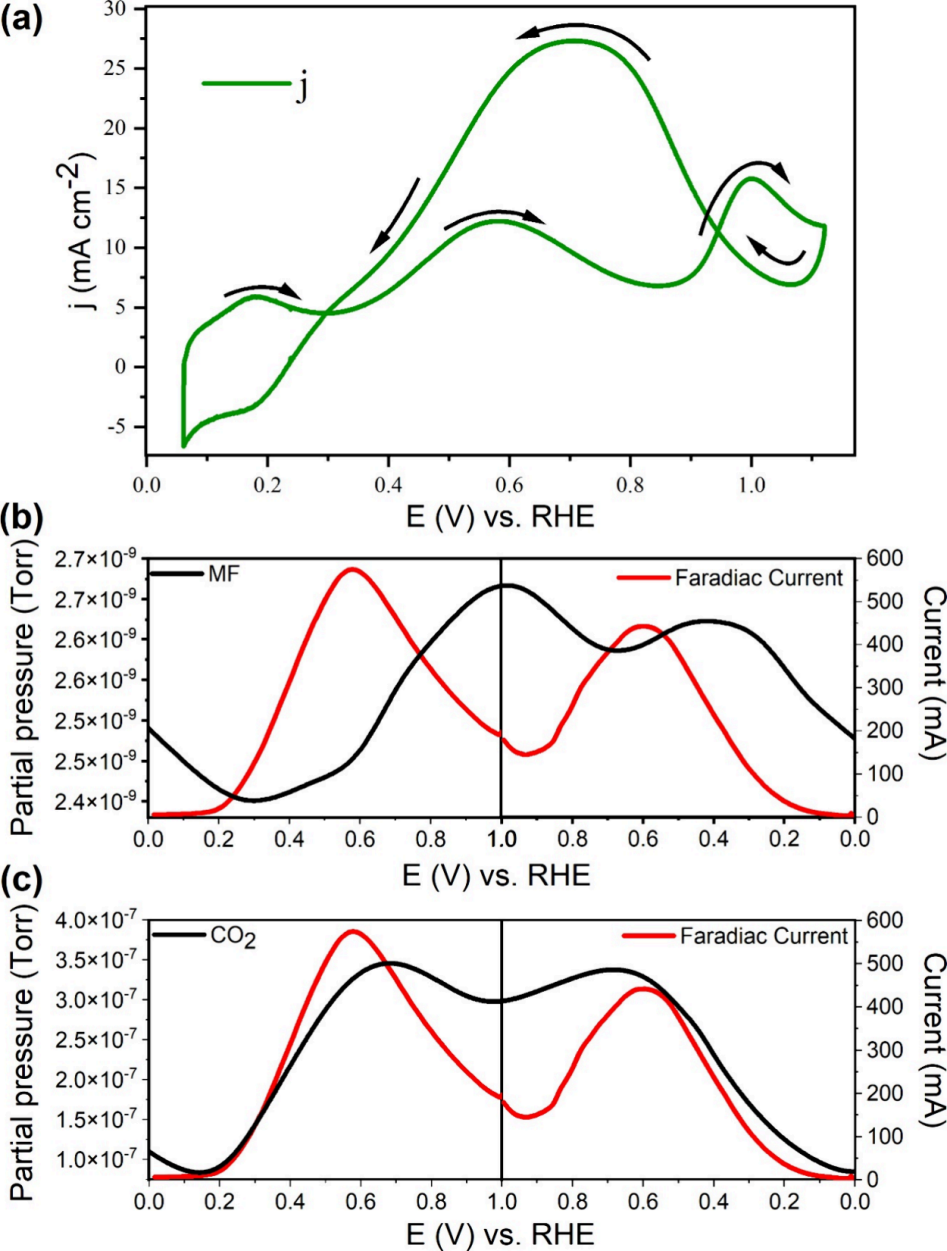
(a) CV recorded in a 0.5 M H_2_SO_4_ electrolyte containing 0.5 M MF using a Pt_3_Pd_3_Sn_2_/C-coated glassy carbon as a working electrode (scan rate 10 mV s^–1^), (b) Mass signal of CO_2_ (*m*/*z* = 44) along with faradaic current, (c) Mass signal of MF (*m*/*z* = 60) with faradaic current during electro-oxidation of methyl formate on Pt_3_Pd_3_Sn_2_ (cell temperature: 70 °C; scan rate: 10 mVs^–1^; 1.0 M MF along with carrier gas (Ar) at the flow rate of 10 mL min^–1^).

Figure 9a shows the CV recorded in a 0.5 M MF solution dissolved in 0.5 M H_2_SO_4_ at 10 mV s^–1^. In the forward scan, two peaks are observed at peak potentials of 0.55 and 0.97 V, with the current density of 12 and 15 mA cm^–2^, respectively. MF contains formate and methanol components, which are oxidized in their respective potential region. The formate component oxidizes at lower potentials than the methanol part.^12^ Hence, the first peak is attributed to the oxidation of the formate component, while the second is attributed to the oxidation of the methanol component and the adsorbed CO. In the reverse scan, an anodic peak is seen at ∼0.7 V, which is attributed to the removal of the oxidized carbonaceous species formed in the forward scan,^43^ as well as the oxidation of oxygen-rich surface from water activation. Therefore, a higher anodic peak in the reverse scan is expected.

#### 3.9.2 Online Electrochemical Mass Spectroscopy

The electro-oxidation of methyl formate on Pt_3_Pd_3_Sn_2_ was examined by online mass spectroscopy (MS). It is known that many fragments are observed in the MS of MF; *m*/*z* = 15, 29, 31, 32, and 60 amu. In this research, the change in the concentration of MF during the electro-oxidation was monitored by following *m*/*z* = 60, because this signal is the only one that does not coincide with any other possible oxidation product: methanol (*m*/*z* = 15, 29, 31, and 32 amu), FA (*m*/*z* = 29, 45, and 46 amu), CO_2_ (*m*/*z* = 44) and formaldehyde (*m*/*z* = 30 amu). The changes in the concentration of other products were monitored by following *m*/*z* = 31 for methanol, *m*/*z* = 46 for FA, *m*/*z* = 44 for CO_2_, and *m*/*z* = 30 amu for formaldehyde. This study was only a qualitative study, not a quantitative one.

First, MFO was examined in the potential range of 0.05–1.00 V (vs RHE). Figures 9b and 9c shows that the oxidation wave of methyl formate starts at 0.15 V. Faradaic current gradually increases with potential, and the peak current was observed at 0.60 V (Figure 9c). The mass signal of CO_2_ (*m*/*z* = 44) also gradually increases with faradaic current and shows a broad peak centered at ∼0.70 V, as depicted in Figure 9b. The peak faradaic current and mass signal do not precisely coincide due to the time delay in the mass signal of CO_2_ in the potentiodynamic mode. The decay in the current above 0.60 V is attributed to the accumulation of OH* on the catalyst surface, which blocks the active sites for the adsorption of fuel. In the cathodic sweep, when the catalyst surface is free from surface oxides, the mass signal of CO_2_ again increases, and a peak was observed at 0.60 V. In this study, MFO was examined at 70 °C, which resulted in a negative shift in onset potential, compared to our previous study.^9^Figure 9c shows the mass signal of MF with an anodic and cathodic sweep. As expected, the initial decrease in the methyl formate mass signal up to 0.30 V was attributed to the adsorption on the catalyst surface, which decreases the free methyl formate available in gaseous form. Once the catalyst surface is completely covered with methyl formate, its mass signal starts increasing again and attains a peak at 1.0 V. At this potential, the catalyst surface is covered with oxide; therefore, methyl formate is not adsorbed on the catalyst surface. In the cathodic sweep, when the catalyst is free from surface oxide, methyl formate adsorption starts again. Methyl formate mass signal (*m*/*z* = 60) showed an opposite trend compared with carbon dioxide (*m*/*z* = 44), which confirms that carbon dioxide is formed by electro-oxidation of the methyl formate.

**Figure 10:**
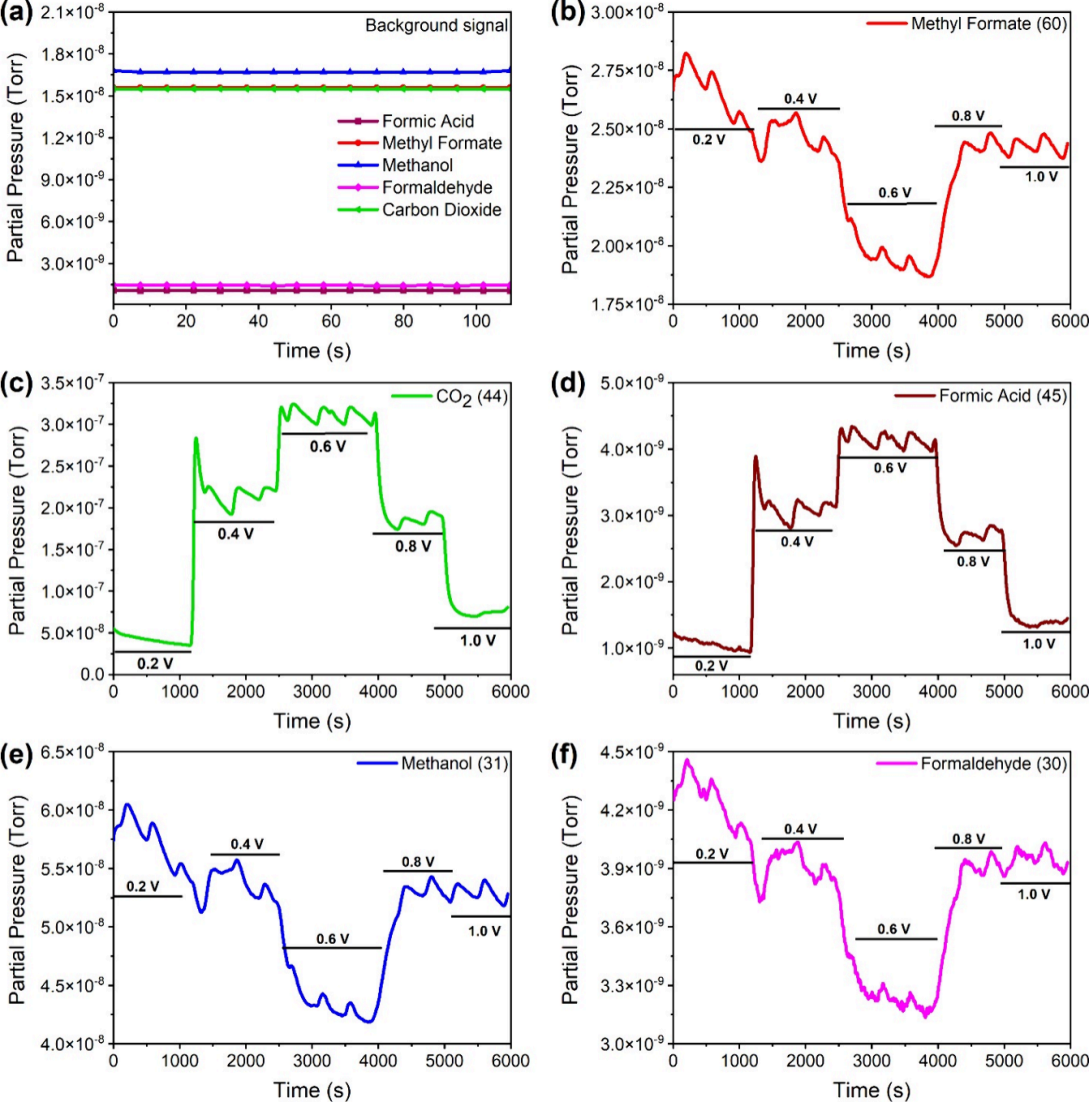
Online mass spectra of (a) background at OCP, (b) MF, (c) carbon dioxide, (d) formic acid, (e) methanol, and (f) formaldehyde recorded in potentiostatic mode on Pt_3_Pd_3_Sn_2_/C (cell temperature, 70 °C; H_2_ flow rate at cathode, 25 mL min^–1^; methyl formate flow rate, along with carrier gas Ar, 10 mL min^–1^).

Methyl formate electro-oxidation was also examined in potentiostatic mode to mitigate the effect of delay in the mass signal and observe changes in the mass signal of intermediate species with applied potential. The background mass spectra were recorded without applying any potential. No change in the mass signal of methyl formate (*m*/*z* = 60), formic acid (*m*/*z* = 45), methanol (*m*/*z* = 31), formaldehyde (*m*/*z* = 30), and CO_2_ (*m*/*z* = 44) was observed at open circuit potential (OCV) (Figure 10a).

Figures 10b–f shows the online gas analysis of MFO products analyzed in the potentiostatic mode. Similarly to MFO in potentiodynamic mode (Figure 9), the potentiostatic mode also showed the same trend in change in the CO_2_ mass signal. The peak of the CO_2_ mass signals (*m*/*z* = 44) reaches its maximal value at 0.60 V (Figure 10c). The mass signal of methyl formate (*m*/*z* = 60)(Figure 10b) exhibited an inverse trend of the CO_2_ mass signal, which confirms that it is consumed in the electrochemical process. It was observed that the mass signal of methyl formate starts decreasing with an increase in potential and reaches a minimum at ∼0.60 V (Figure 10b), the potential at which the CO_2_ peak was observed. A steep decline of the CO_2_ signal and an increase in methyl formate signal above 0.60 was ascribed to the deactivation of the catalyst due to the formation of surface oxides. It has been reported that the formate/formic acid part of MF preferentially oxidizes in the low potential region (<0.60 V), whereas methanol oxidizes through the indirect pathway at higher potentials (>0.60 V) on platinum-based catalysts.^10,17^ The change in formic acid (Figure 10d) signal with potential is the same as the change in the signal of CO_2_ (*m*/*z* = 44), confirming that it is formed electrochemically in the same reaction path in which CO_2_ is formed, and not in a lower potential, which is consistent with the DFT studies.

Since the observed mass signals of methanol (*m*/*z* = 31) (Figure 10e) and formaldehyde (*m*/*z* = 30) (Figure 10f) follow the same trend as MF (Figure 10e), they are attributed to the fragmentation of MF in the MS. The oxidation process starts with dehydrogenation followed by the scission of the C–O bond, and the onset of CO_2_ evolution was also observed at a very low potential (<0.20 V). During dehydrogenation, CO* is formed. According to the DFT calculations, CO* is the main poisoning intermediate and rate-limiting step in MFEO. DFT suggests its removal by adsorbed oxygens on the bridge site of the Sn–Pd (Figure S4a), it enables the formation of CO_2_ at very low potentials (0.4 V), but as the electrode potential increases (0.6 V), the dehydrogenation of the water molecule to form OH* is facilitated, and oxidative removal of CO* by OH* becomes also possible; therefore, the CO_2_ mass signal starts increasing, consistent with DFT results. Since there is no simultaneous increase in the methanol and MF signals during the oxidation, the hydrolysis on Pt_3_Pd_3_Sn_2_ is excluded.

## 4 Conclusion

The periodic boundary density functional theory was used to elucidate the thermochemical and kinetic aspects of MF electro-oxidation on the Pt_3_Pd_3_Sn_2_ (111) surface. Parallelly, MFEO was studied experimentally, and OE-MS was used to validate the intermediates and products observed during MFEO.

The DFT calculations confirmed that the most stable adsorption is the trans-configuration of MF, adsorbed via Pt and Pd atoms, while Sn is not directly involved in MF adsorption. This finding was further confirmed by an electronic structure calculation. The synergy between Pt and Pd facilitates a preferred active site for initiating MF oxidation through O–H bond scission, while Sn plays an essential role in providing the active site for the cleavage of the CH_3_O* and CO* bond from CH_3_OCO*. By considering the reaction energies and activation barriers for all possible reactions in the MFEO, the favored oxidation pathway is CH_3_OCHO* → CH_3_OCO* → CH_3_O* + CO* → CH_2_O* → CHO* → CO* → CO_2_*, → CO_2_ (aq). This energy path necessitates a minimum energy of 0.14 eV to initiate MF oxidation. This significantly low value is supported by the experimental results, showing that the oxidation wave of methyl formate starts at 0.15 V (at 70 °C). The derived free-energy diagram via DFT calculations suggests that the preferred oxidation pathway for CH_3_OCHO to CO_2_ is the indirect mechanism in which CO is formed as an intermediate. The presence of tin oxides facilitates the removal of CO* as CO_2_ via oxygenation. Under experimental conditions, in which there is no source of oxygen, the removal of CO* occurs through the reaction with OH* at the Pd site. At higher applied potentials, the removal of CO* with OH* becomes feasible, as predicted by the DFT calculations and shown experimentally.

The DFT results, supported by OE-MS, indicate that the hydrolysis of MF on the Pt_3_Pd_3_Sn_2_ (111) alloy, prior to the MFEO is not preferred, although the formation of methanol is plausible via another route, from the CH_3_O intermediate.

This research points out the advantages of MF as a fuel in PEMFCs. Among the three SOMs studied in this manuscript, MF, methanol, and formic acid, the activation energy for the first bond breaking, which initiates the whole process, is significantly lower for MF (0.13 eV) than for formic acid (0.45 eV) and the highest value is obtained for methanol (0.61 eV). These results indicate that MF is the preferred fuel for electro-oxidation compared to methanol and FA when the electrocatalyst is Pt_3_Pd_3_Sn_2_ (111) alloy.

By conducting both theoretical and experimental research, we have gained a deep understanding of the catalytic efficiency of a trimetallic catalyst, which provides a variety of adsorption sites and selectivity. Thus, this research is expected to affect the usage of metallic alloy catalysts for energy conversion technologies.

## Supporting Information Available

Methodology of the computation, together with tables with additional energy results, Bader charge analysis, and thermochemical reaction mechanism for the MFEO; figures describing the various steps in the energy path PDF)
