# Supplementary material for: Trimetallic Alloys as an Electrocatalyst for Fuel Cells: The Case of Methyl Formate on Pt3Pd3Sn2
Source: ACS Appl Mater Interfaces. 2024 Oct 16;16(43):58573–86. doi: 10.1021/acsami.4c11282 (PMC11533157; doi:10.1021/acsami.4c11282)
Supplement: Supplementary file 1 — am4c11282_si_001.pdf [file am4c11282_si_001.pdf]

Supporting Information

Tri-Metallic Alloys as an Electro-Catalyst for Fuel Cells – The Case of Methyl Formate on $Pt_3Pd_3Sn_2$

*Radhey Shyam Yadav[a], Diwakar Kashyap[a], Itay Pitussi[a], Medhanie Gebremedhin Gebru[a], Hanan Teller[a], Alex Schechter[a,b], Haya Kornweitz[a],**

[a] *Department of Chemical Sciences, Ariel University, Ariel, 40700, Israel*

[b] *Research and Development Centre for Renewable Energy, New Technology Centre, University of West Bohemia, 301 00 Pilsen, Czech Republic.*

*Email: hayak@ariel.ac.il

Methodology:

The aqueous-phase free energies are calculated relative to the DFT- derived energies of H_2O(aq), CO_2(aq), and H_2(aq). For example, the free energy of MF is calculated from the reaction:

$2CO_2(aq) + 4\ H_2(aq) \longrightarrow CH_3OCHO(aq) + 2\ H_2O(aq)$ ……………………..…..(1)

From which the free energy of CH_3OCHO is calculated as[13])

$$\Delta G_{CH_3OCHO} = (E_{CH_3OCHO} - TS_{CH_3OCHO} + ZPE_{CH_3OCHO}) + 2(E_{H_2O} - TS_{H_2O} + ZPE_{H_2O}) - 2(E_{CO_2} - TS_{CO_2} + ZPE_{CO_2}) - 4(E_{H_2} - TS_{H_2} + ZPE_{H_2})$$
…………………………………………………….…………………………(2)

Where E is the total energy of species calculated from DFT, T is the standard temperature (298k), S is the entropy and ZPE is the calculated zero-point energy for the species. The calculation for the free energies of other species is done similarly. When calculating the free energy of adsorbed species, the total energy of a species is taken relative to the gas phase and clean surface, while the entropy and zero-point energy are calculated for the adsorbed species (*). An example of the free energy change of a reaction involving surface intermediates is:

$2CO_2(aq) + 4\ H_2(aq) + * \longrightarrow CH_3OCHO^* + 2\ H_2O(aq)$ ………………………..…(3)

From which the free energy of CH_3OCHO^* is calculated as:

$$\Delta G_{CH_3OCHO^*} = (E_{CH_3OCHO^*} - TS_{CH_3OCHO^*} + ZPE_{CH_3OCHO^*}) + 2\ (E_{H_2O} - TS_{H_2O} + ZPE_{H_2O}) - 2(E_{CO_2} - TS_{CO_2} + ZPE_{CO_2}) - 4(E_{H_2} - TS_{H_2} + ZPE_{H_2}) - (E_{surface} - TS_{surface} + ZPE_{surface})$$

……………………………………………………………………………...(4)

Where * is showing the adsorbed species, $E_{surface}$ is the total energy of the clean surface, and the rest of the terms are defined above.

The free energy of adsorption (G) for each species X is defined as:

$$G_{ads}(X) = (E_X^* + ZPE_X^* - TS_X^*) - (E_{slab} + ZPE_{slab} - TS_{slab}) - (E_X + ZPE_X - TS_X) \ldots\ldots(5)$$

Where, E^*_X represents the total energy of the adsorbate X on slab system, E_{slab} is the total energy of the clean slab, and E_X is the energy of the adsorbate in the aqueous-phase. The term ZPE*, ZPE_{slab} and ZPE, are the zero-point energy for the total adsorbed system, clean slab, and adsorbate, while T, S.*, $S_{slab,}$ and S are the temperature and entropy for the adsorbed system, clean surface, and gas-phase species, respectively. According to this equation, a negative value of E_{ads} signifies exergonic adsorption.

The activation energy is defined as the difference between the energy of the transition state and the reactants.

$$E_a = (E_{ts} + ZPE_{ts} - TS_{ts}) - (E_r + ZPE_r - TS_r) \ldots\ldots(6)$$

Where E_a, E_{ts} and E_r are the activation energy, energy of transition state and energy of reactant respectively, other terms are defined above.

The energy of a reaction, e.g.: A + B → C + D, is defined as:

$$\Delta G^0 = G_C + G_D - G_A - G_B \ldots\ldots(7)$$

Where $G_{A,}$ $G_{B,}$ $G_{C,}$ and G_D are defined by equation S2.

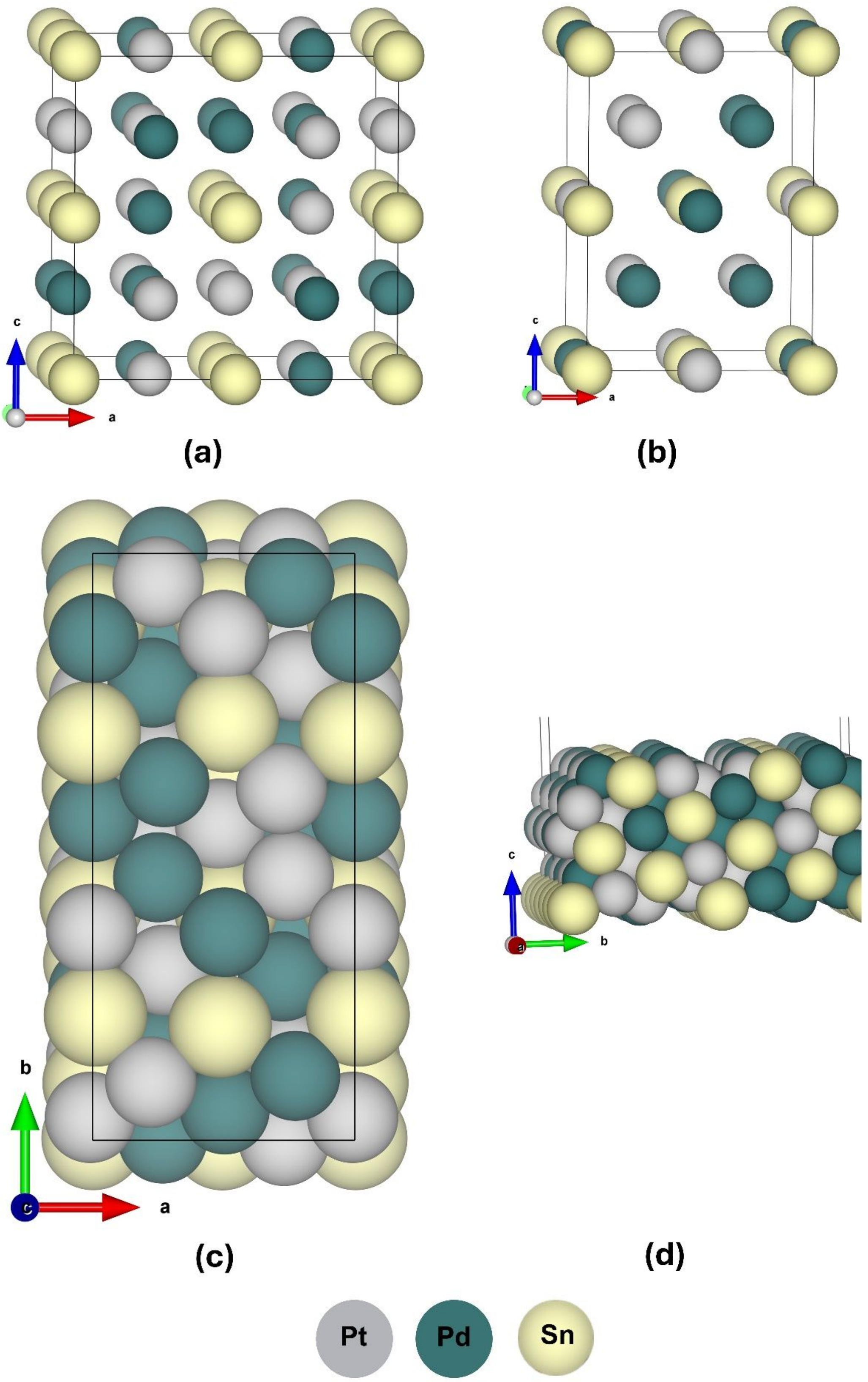

Figure S1: **(a)** Modified bulk unit cell of $Pt_3Pd_3Sn_2$ alloy, **(b)** Primitive unit cell of modified bulk, **(c)** Top view of 111 surface of $Pt_3Pd_3Sn_2$ alloy **(d)** Side view of 111 surface of $Pt_3Pd_3Sn_2$ alloy.

Table S1: The electronic adsorption energy of all the species involved in MFEO.

SN	Species	Top site				Bridge site				Other
		Pd	Pt	Sn	Pt-Pd	Pt-Sn	Pd-Sn	Pd-Pd	Pt-Pd	
1	CH_3OCHO (trans)	-0.84<br>-0.55*	-0.55	-0.53	-	-	-	-	-	-0.53<br>(top)<br>(Pd-Sn)
	CH_3OCHO (cis)	-0.79	-0.73	-	-058	--	-	-0.56	-	-
2	CH_3OCO (trans)	-2.47	-2.91	-1.36<br>Sn-Pt<br>(top)	-	-	-	-	-	-1.09<br>(Sn-Pd)<br>(top)
	CH_3OCO (cis)	-2.46	-	-	-	-2.94	-	-	-	-
3	CH_2OCHO	-	-	-	-3.14	-	-	-	-	-2.65<br>(Pd-Pt)
4	CHOCHO	-2.53	-3.29	-	-3.20			-3.56	-	-
5	CH_3O	-2.36	-2.66	-2.61	-	-2.77<br>-2.62	-2.83<br>-2.76	-	-	-
6	CH_2O	-0.86	-0.93	-	-	-0.89	-0.79	-	-0.48	-
7	CHO	-2.39	-2.74	-	-	-	-	-		-
8	CO	-1.51	-2.03	-	-	-	-	-	-2.52	-
9	H_2O	-0.27	-0.42	-0.27	-	-	-0.34	-		-
10	CH_3OH	-0.73	-0.64	-0.52	-	-0.55	-0.52	-	-0.72	-
11	CH_2OH	-0.90	-2.47	-	-	-2.46	-1.58	-		-
12	CHOH	-2.36	-2.99	-1.05	-	-	-	-		-
13	CH_3	-2.36	-2.76	-2.16	-	-	-	-		-
14	CH_2	-3.26	-3.92	-	-	-	-	-	-5.26	-
15	H	-3.25	-3.81	-	-	-	-	-	-4.08	-
16	O	-4.80	-5.49	-	-	-	-6.13	-		-6.00<br>(hollow<br>)
21	COH	-2.09	-	-	-	-	-	-		-
22	CO_2	-0.06	-0.09	-	-	-	-	-		-
23	O_2*	--	-1.80	-	-	-2.14<br>-1.64	-1.89	-1.82	-2.35	-2.22<br>(hollow<br>)

Table S2: Bader charge analysis for each atom of MF molecule on the surface of $Pt_3Pd_3Sn_2(111)$.

**CH_3OCHO (trans)**		**CH_3OCHO (cis)**	
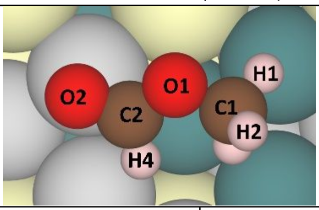		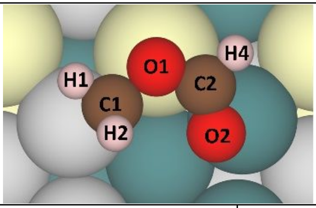	
**Atom in CH_3OCHO**	**Q (e)**	**Atom in CH_3OCHO**	**Q (e)**
C1	0.05	C1	0.03
C2	0.12	C2	0.07
O1	0.04	O1	0.00
O2	-0.07	O2	-0.04
H1	0.02	H1	-0.02
H2	-0.03	H2	-0.01
H3	-0.03	H3	0.00
H4	0.00	H4	-0.02
**Total**	**0.10**	**Total**	**0.01**

Table S3: D-band centre (ε_d), p-band centre (ε_p), s-band centre (ε_s), difference in (ε_d and ε_p)(eV) and difference in (ε_d and ε_s) (eV) for both trans and cis configuration.

	ε_d (eV)		ε_p (eV)		ε_s (eV)	ε_d - ε_p (eV)		ε_d - ε_p (eV)		ε_d - ε_s (eV)	
	Pt	Pd	C	O		(Pt-C)	(Pd-C)	(Pt-O)	(Pd-O)	(Pt-H)	(Pd-H)
Trans	-2.39	-2.14	-2.88	-3.38	-4.95	0.49	0.73	0.99	1.24	2.56	2.81
Cis	-2.38	-2.14	-2.97	-3.35	-5.04	0.59	0.83	0.97	1.21	2.66	2.90

Table S4: Thermochemical reaction energy ΔG (eV) for all the possible steps involved in the MFEO.

SN	Reactions	ΔG (eV)
1	$CH_3OCHO^* \rightarrow CH_3OCO^* + H^+ + e^-$	-0.24
2	$CH_3OCHO^* \rightarrow CH_2OCHO^* + H^+ + e^-$	0.08
3	$CH_3OCHO^* \rightarrow CH_3O^* + OCH^*$	-0.14
4	$CH_3OCHO^* \rightarrow CH_3OH^* + CO^*$	-1.41
5	$CH_3OCO^* \rightarrow CH_2OCO^* + H^+ + e^-$	0.87
6	$CH_3OCO^* \rightarrow CH_3^* + CO_2^*$	-0.23
7	$CH_3OCO^* \rightarrow CH_3O^* + CO^*$	-0.60
8	$CH_3OCO^* \rightarrow CH_2O^* + CO^* + H^+ + e^-$	-0.37
9	$CH_3OCO^* \rightarrow CH_2^* + CO_2^* + H^+ + e^-$	0.01
10	$CH_2OCHO^* \rightarrow CHOCHO^* + H^+ + e^-$	1.04
11	$CH_2OCHO^* \rightarrow CH_2OCO^* + H^+ + e^-$	0.55
12	$CH_2OCHO^* \rightarrow CH_2O^* + CHO^*$	0.01
13	$CH_2OCO^* \rightarrow CHOCO^* + H^+ + e^-$	1.17
14	$CH_2OCO^* \rightarrow CHO^* + CO^* + H^+ + e^-$	-1.50
15	$CH_2OCO^* \rightarrow CH_2^* + OCO^*$	-0.86
16	$CHOCO^* \rightarrow 2CO^* + H^+ + e^-$	-3.39
17	$CHOCO^* \rightarrow CHO^* + CO^*$	-2.68
18	$CH_3O^* \rightarrow CH_2O^* + H^+ + e^-$	0.23
19	$CH_2O^* \rightarrow CHO^* + H^+ + e^-$	-0.25
20	$CHO^* \rightarrow CO^* + H^+ + e^-$	-0.71
21	$CHO^* + OH^* \rightarrow HCOOH^*$	-0.08
22	$HCOOH^* \rightarrow HCOO^* + H^+ + e^-$	0.28
23	$HCOOH^* \rightarrow COOH^* + H^+ + e^-$	0.06
24	$COOH^* \rightarrow CO_2^* + H^+ + e^-$	0.69
25	$HCOO^* \rightarrow CO_2^* + H^+ + e^-$	0.47
26	$CH_3^* + OH^* \rightarrow CH_3OH^*$	0.49
27	$CH_3OH^* \rightarrow CH_2OH^* + H^+ + e^-$	0.41
28	$CH_3OH^* \rightarrow CH_3O^* + H^+ + e^-$	0.57
29	$CH_2OH^* \rightarrow CHOH^* + H^+ + e^-$	0.29
30	$CH_2OH^* \rightarrow CH2O^* + H^+ + e^-$	0.38
31	$CHOH^* \rightarrow COH^* + H^+ + e^-$	0.49
32	$CHOH^* \rightarrow CHO^* + H^+ + e^-$	-0.16
33	$COH^* \rightarrow CO^* + H^+ + e^-$	-1.36
34	$CH_2^* + OH^* \rightarrow CH_2OH^*$	0.60
35	$CH_3^* \rightarrow CH_2 + H^+ + e^-$	0.24
36	$CO^* + OH^* \rightarrow COOH^*$	0.68
37	$CO^* + OH^* \rightarrow CO_2^* + H^+ + e^-$	1.37
38	$CO^* + O^* \rightarrow CO_2^*$	0.14
39	$H_2O^* \rightarrow OH^* + H^+ + e^-$	0.52

40	$OH^* \rightarrow O^* + H^+ + e^-$	1.23
41	$CH_3OCHO^* + H_2O^* \rightarrow HCOOH^* + CH_3OH^*$	-0.27
42	$CH_3OCHO + H_2O \rightarrow HCOOH + CH_3OH$	0.09

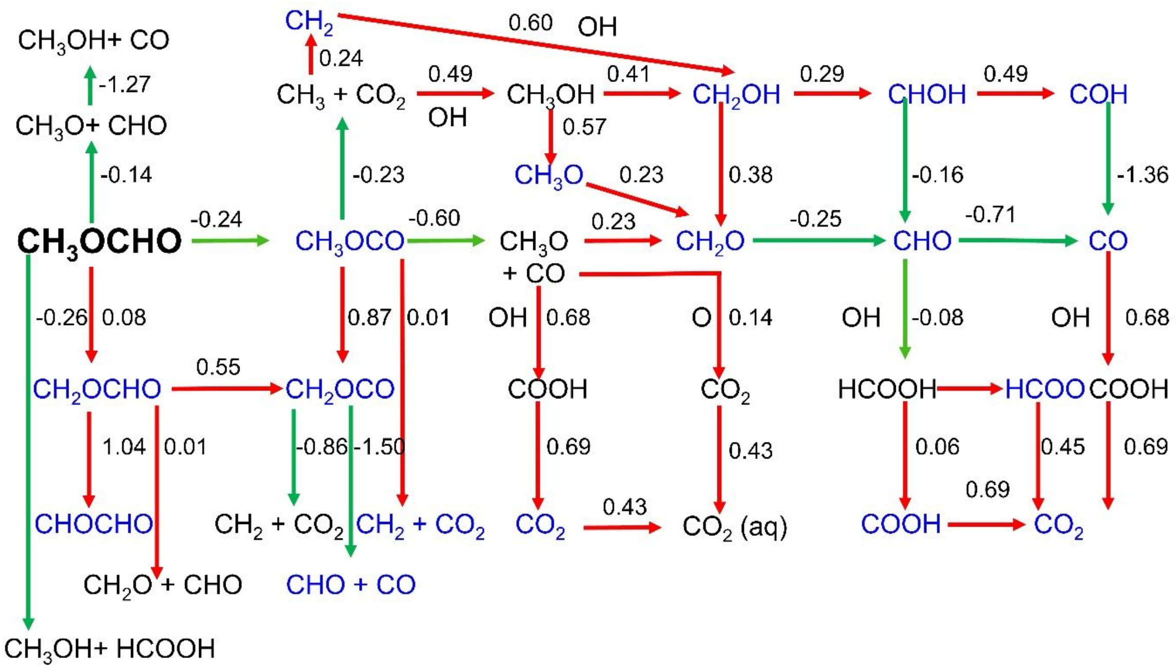

Scheme S1: Thermochemical reaction mechanism for the MFEO. Green and red color arrows represent the exergonic and endergonic reactions, respectively. The number with black color represents the reaction energy. Products with blue and black color are showing the proton-electron transfer step and decomposition reactions, respectively. All the values marked on the arrows are the free energy of the reaction step given in eV.

Table S5: The activation barrier (E_a) and reaction energy (ΔG) for the dehydrogenated and deprotonated steps involved in MFEO.

SN	Reactions	E_a (eV)	X*→ Y* +Z* (Decomposition)	X→ Y* + H^+ + e^- (Deprotonation)
			ΔG (ev)	ΔG (ev)
1	$CH_3OCHO^* \rightarrow CH_3OCO^* + H^+ + e^-$	0.13	-0.96	-0.24
2	$CH_3OCHO^* \rightarrow CH_2OCHO^* + H^+ + e^-$	0.57	0.07	0.78
3	$CH_3OCHO^* \rightarrow CH_3O^* + CHO^*$	0.71	0.26	-
4	$CH_3OCHO^* \rightarrow CH_3OH^* + CO^*$	0.71	-0.91	-
5	$CH_3OCO^* \rightarrow CH_3^* + CO_2^*$	1.85	-0.23	-
6	$CH_3OCO^* \rightarrow CH_3O^* + CO^*$	0.50	-0.08	-
7	$CH_3O^* \rightarrow CH_2O^* + H^+ + e^-$	0.13	-0.70	0.02
8	$CH_2O^* \rightarrow CHO^* + H^+ + e^-$	0.00	-0.93	-0.22
9	$CHO^* \rightarrow CO^* + H^+ + e^-$	0.07	-0.90	-0.18
10	$CHO^* + OH^* \rightarrow HCOOH^*$	1.22	-0.08	-
11	$HCOOH^*(Pd) \rightarrow HCOO^* + H^+ + e^-$	0.45	-0.44	0.28
12	$HCOOH^*(Pd) \rightarrow COOH^* + H^+ + e^-$	1.37	-0.23	0.49
13	$COOH^*(Pd) \rightarrow CO_2^* + H^+ + e^-$	0.46	-0.46	0.26
14	$HCOO^* \rightarrow CO_2^* + H^+ + e^-$	0.25	-0.25	0.47
15	$CH_3OH^* \rightarrow CH_3O^* + H^+ + e^-$	0.61	-0.12	0.59
16	$CH_3OH^* \rightarrow CH_2OH^* + H^+ + e^-$	0.81	-0.09	0.62
17	$CH_3OH^* \rightarrow CH_3O^* + H^+ + e^-$	0.61	-0.12	0.59
18	$CH_2OH^* \rightarrow CHOH^* + H^+ + e^-$	0.16	-0.78	-0.06
19	$CH_2OH^* \rightarrow CH_2O^* + H^+ + e^-$	0.33	-0.72	-0.01
20	$CHOH^* \rightarrow COH^* + H^+ + e^-$	0.55	-0.23	0.49
21	$CHOH^* \rightarrow CHO^* + H^+ + e^-$	0.09	-0.88	-0.16
22	$COH^* \rightarrow CO^* + H^+ + e^-$	0.13	-1.55	-0.83
23	$CO^*(Pt) + OH^* \rightarrow COOH^*(Pt)$	1.13	0.33	-
24	$COOH^*(Pt) \rightarrow CO_2^* + H^+ + e^-$	0.51	-0.03	0.69
25	$CO^*(Pd) + OH^* \rightarrow COOH^*(Pd)$	0.96	0.59	-
26	$CO^*(Pd) + O^* \rightarrow CO_2^*$	0.82	-0.37	-
27	$CO^*(Pt) + O^* \rightarrow CO_2^*$	0.74	-0.19	-
28	$CH_3OCHO^* \rightarrow CH_3OH^* + CO^*$	0.71	-0.91	-

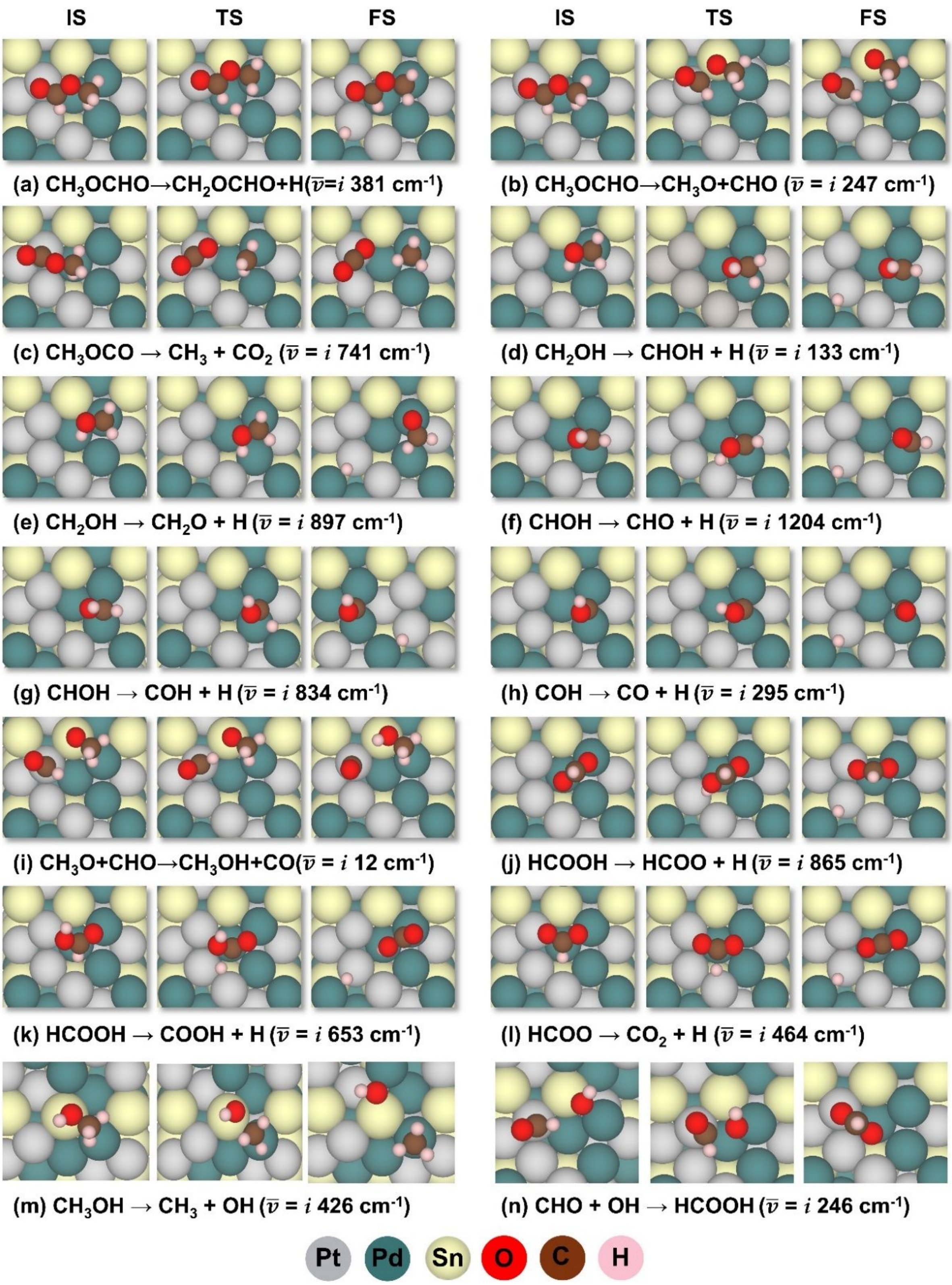

Figure S2: (a-n) The geometries of the initial state (IS), final state (FS), and transition state (TS) involved in the activation barrier calculation for the various reactions involved in MFEO.

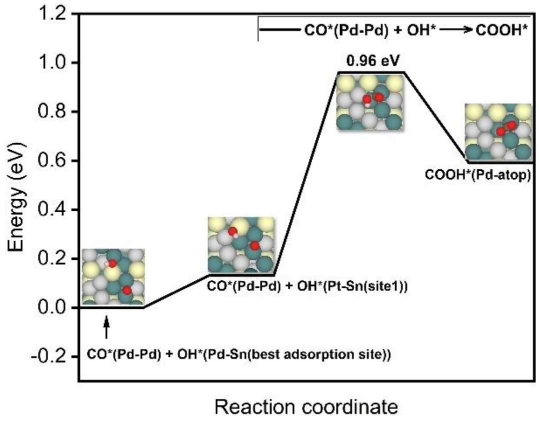

a. CO*(Pd-Pd) + OH* → COOH*

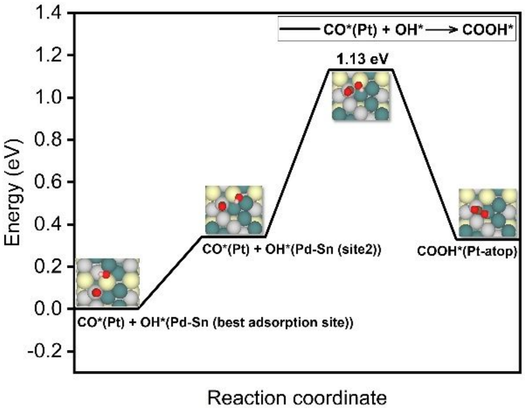

b. CO*(Pt) + OH* → COOH*

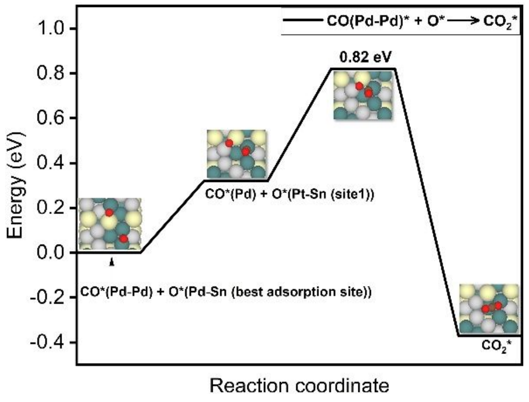

c. CO*(Pd-Pd) + O* → CO_2*

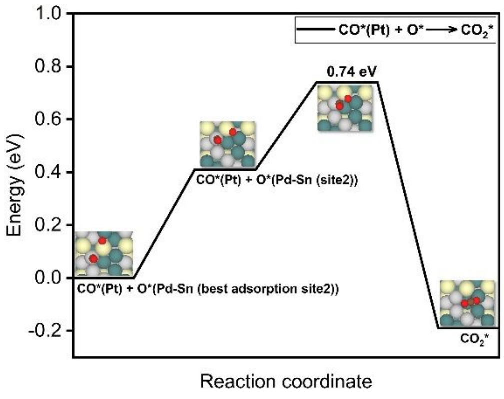

d. CO*(Pt) + O* → CO_2*

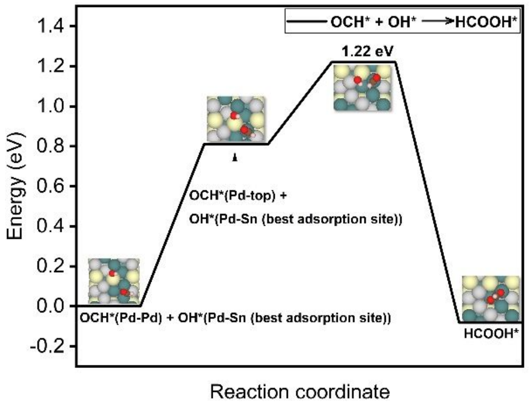

e. CHO*(Pd-Pd) + OH* → HCOOH (Pd)*

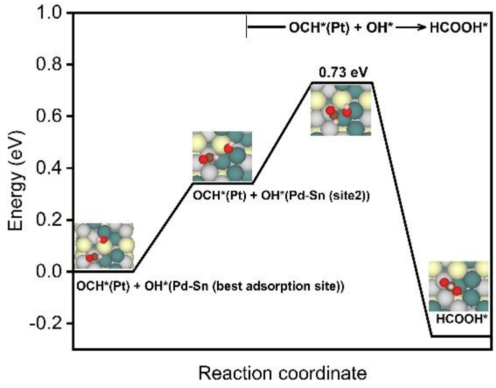

f. CHO*(Pt) + OH* → HCOOH (Pt)*

Figure S3: (a-f) Potential energy diagram for the two-step processes for TS in MFEO.

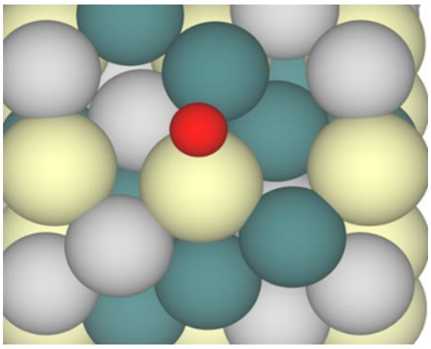
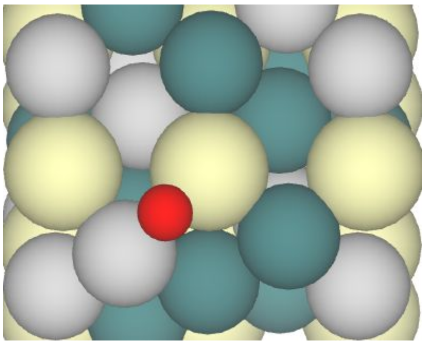
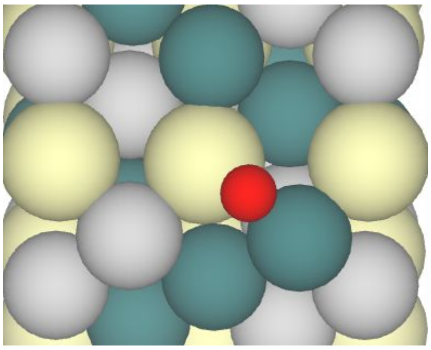

(a) O (Pd-Sn (Best adsorption))
(E_{ads} = -6.13 eV)

(b) O (Pt-Sn (site1))
(Eads = -6.07 eV)

(c) O (Pd-Sn (site2))
(Eads = -5.73 eV)

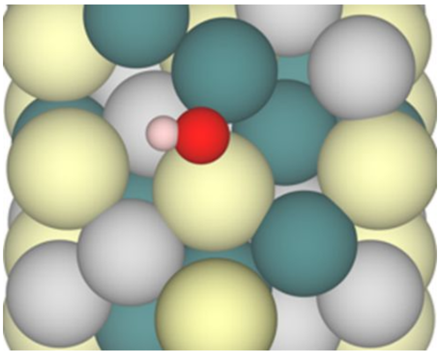
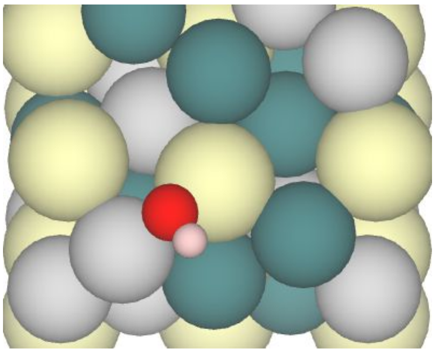
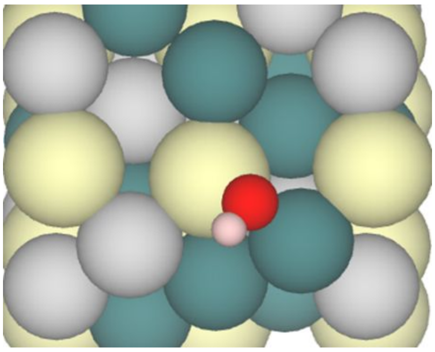

(d) OH (Pd-Sn (Best adsorption))
(Eads = -3.58 eV)

(e) OH (Pt-Sn (site1))
(Eads = -3.45 eV)

(f) OH (Pd-Sn (site2))
(Eads = -3.24 eV)

Figure S4: Possible adsorption site of: **(a-c)** Oxygen, **(d-f)** OH, involved in two-step processes in TS calculation.

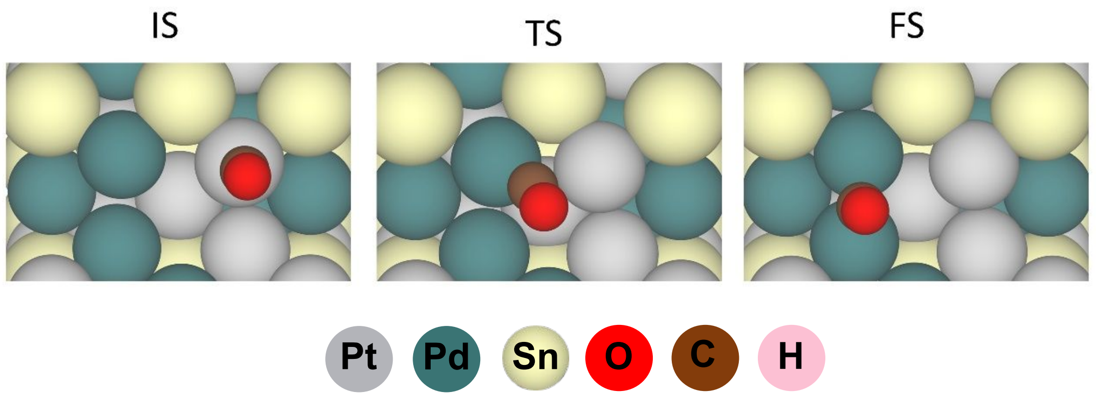

Figure S5: The IS, TS, and FS for the transition state calculation of CO diffusion from the top of the Pt site to the Pd-Pd bridge site.
